# Data-driven reverse engineering of signaling pathways using ensembles of dynamic models

**DOI:** 10.1371/journal.pcbi.1005379

**Published:** 2017-02-06

**Authors:** David Henriques, Alejandro F. Villaverde, Miguel Rocha, Julio Saez-Rodriguez, Julio R. Banga

**Affiliations:** 1 Bioprocess Engineering Group, Spanish National Research Council, IIM-CSIC, Vigo, Spain; 2 Centre of Biological Engineering, University of Minho, Braga, Portugal; 3 Joint Research Center for Computational Biomedicine, RWTH-Aachen University, Aachen, Germany; 4 European Molecular Biology Laboratory, European Bioinformatics Institute (EMBL-EBI), Wellcome Trust Genome Campus, Hinxton, United Kingdom; University of Pennsylvania, UNITED STATES

## Abstract

Despite significant efforts and remarkable progress, the inference of signaling networks from experimental data remains very challenging. The problem is particularly difficult when the objective is to obtain a *dynamic* model capable of predicting the effect of novel perturbations not considered during model training. The problem is ill-posed due to the nonlinear nature of these systems, the fact that only a fraction of the involved proteins and their post-translational modifications can be measured, and limitations on the technologies used for growing cells *in vitro*, perturbing them, and measuring their variations. As a consequence, there is a pervasive lack of identifiability. To overcome these issues, we present a methodology called SELDOM (enSEmbLe of Dynamic lOgic-based Models), which builds an ensemble of logic-based dynamic models, trains them to experimental data, and combines their individual simulations into an ensemble prediction. It also includes a model reduction step to prune spurious interactions and mitigate overfitting. SELDOM is a data-driven method, in the sense that it does not require any prior knowledge of the system: the interaction networks that act as scaffolds for the dynamic models are inferred from data using mutual information. We have tested SELDOM on a number of experimental and *in silico* signal transduction case-studies, including the recent HPN-DREAM breast cancer challenge. We found that its performance is highly competitive compared to state-of-the-art methods for the purpose of recovering network topology. More importantly, the utility of SELDOM goes beyond basic network inference (i.e. uncovering *static* interaction networks): it builds *dynamic* (based on ordinary differential equation) models, which can be used for mechanistic interpretations and reliable dynamic predictions in new experimental conditions (i.e. not used in the training). For this task, SELDOM’s ensemble prediction is not only consistently better than predictions from individual models, but also often outperforms the state of the art represented by the methods used in the HPN-DREAM challenge.

## Author summary

Signaling pathways play a key role in complex diseases such as cancer, for which the development of novel therapies is a difficult, expensive and laborious task. Computational models that can predict the effect of a new combination of drugs without having to test it experimentally can help in accelerating this process. In particular, network-based dynamic models of these pathways hold promise to both understand and predict the effect of therapeutics. However, their use is currently hampered by limitations in our knowledge of the underlying biochemistry, as well as in the experimental and computational technologies used for calibrating the models. Thus, the results from such models need to be carefully interpreted and used in order to avoid biased predictions. Here we present a procedure that deals with this uncertainty by using experimental data to build an ensemble of dynamic models. The method incorporates steps to reduce overfitting and maximize predictive capability. We find that by combining the outputs of individual models in an ensemble it is possible to obtain a more robust prediction. We report results obtained with this method, which we call SELDOM (enSEmbLe of Dynamic lOgic-based Models), showing that it improves the predictions previously reported for several challenging problems.

This is a *PLOS Computational Biology* Methods paper

## Introduction

Inferring the molecular circuits of the cell from experimental data is a fundamental question of systems biology. In particular, the identification of signaling and regulatory networks in healthy and diseased human cells is a powerful approach to unravel the mechanisms controlling biological homeostasis and their malfunctioning in diseases, and can lead to the development of novel therapies [1, 2]. Given the complexity of these networks, these problems can only be addressed effectively combining experimental techniques with computational algorithms. Such network inference (or reverse engineering) efforts [3] have been largely developed for gene regulation [4, 5], and to a lesser extent for signal transduction [1]. Extensive work has been published on the inference of molecular circuits, either as static networks—that is, recovering only the topology of interactions—[4–6] or as dynamical systems [7, 8]. It can be beneficial to consider network inference in conjunction with the prediction of data for new conditions, since a precise topology should help in the generation of high quality predictions, and the inability of a model topology to describe a given set of experiments suggests that the model is in some sense wrong or incomplete.

Signal transduction is a highly dynamic process, and the identification and analysis of the underlying systems requires dynamical data of the status of its main players (proteins) upon perturbation with ligands and drugs. These experiments are relatively complex and expensive, and there is a trade-off between coverage and throughput [2] that often makes the problem ill-posed, leading to identifiability issues. The problem of handling parametric and structural uncertainty in dynamic models of biological systems has received great attention in systems biology and biotechnology [9–12]. Inference and identification methods can be used to find families of dynamic models compatible with the available data, but in general these models will still suffer from lack of identifiability in a certain degree [3].

Ensemble modeling can be used to improve the predictive capabilities of models, helping to overcome the fundamental difficulties associated with lack of structural and/or practical identifiability. The usage of ensemble methods is widespread in fields such as machine learning [13], bioinformatics [14], and weather forecasting, but not so much in computational systems biology, although it has been successfully applied in the context of regulatory [15, 16], metabolic [17, 18], and signaling [19] networks. Although there is no universally agreed explanation of the success of ensemble methods as classifiers in machine learning [20], it has been shown that they can improve generalization accuracy by decreasing variance [21], bias [22] or both [23], and the reasons for this are relatively well understood [13]. A common approach for building an ensemble is to train a number of so-called base learners in a supervised manner, using data re-sampling strategies. An example of the application of such methods in biology can be found in [24], where the inference of gene regulatory networks is formulated as a feature selection problem, and regression is performed using tree-based ensemble methods. This approach was recently extended to accommodate dynamics [25]. Xing et al [26] used ensemble simulations of causal genetic networks to predict genes involved in rheumatoid arthritis. They showed that the use of ensembles allows for quantitative prediction of the effects of perturbation, adding robustness to predictions by accounting for uncertainty in network topology. Besides robustifying predictions, another task in which ensembles can be helpful is in the elucidation of insufficiently characterized circuits. In this context, Kuepfer et al [19] showed how to use ensembles to unravel operating principles in signaling pathways. They created an ensemble of plausible models of the target of rapamycin (TOR) pathway in S. cerevisiae, in which the different topologies accounted for uncertainties in network structure: each member of the ensemble extended a core model by including an additional reaction. By clustering the models according to their training errors, they determined the common features shared by those that better reproduced the experimentally observed behaviour. In this way, a new factor was proposed as the key signaling mechanism. Ensembles of dynamic systems have been used for many years in weather forecasting. In that community, sets of simulations with different initial conditions (ensemble modeling) and/or models developed by different groups (multi-model ensemble) are combined to deliver improved forecasts [27, 28]. In the context of metabolism, Lee et al [29] have shown how to use ensembles to assess the robustness of non-native engineered metabolic pathways. Using the ensemble generation method proposed in [18], a sampling scheme is used to generate representative sets of parameters/fluxes vectors, compatible with a known stoichiometric matrix. This approach is based on the fact that this problem is typically underdetermined, *i.e.* there are more reactions/fluxes than metabolites. Thus, model ensembles may be generated by considering all theoretically possible models, or a representative sample of it. The use of an ensemble composed of all models compatible with the data has been applied to gene regulatory [15] and signal transduction networks [30].

If the model structure is unknown, the ensemble generation needs to be completely data-driven. A common approach for inferring network structures from data is to use estimations of information-theoretic measures, such as entropy and mutual information. There is a plethora of methods for inferring static networks, including correlation, Bayesian inference, and information theory metrics [3, 31–33]. We have focused on information-theoretic approaches because of their good properties regarding handling of nonlinear interactions (which are common in signalling pathways) and scalability. For a recent review of information-theoretic methods, see [6]. The central concept in information theory is entropy, a measure of the uncertainty of a random variable [34]. Mutual information, which can be obtained as a function of the entropies of two variables, measures the amount of information that one random variable provides about another. The mutual information between pairs of variables can be estimated from a data-set, and this can be used to determine the existence of interactions between variables, thus allowing the reverse engineering of network structure. For early examples of this approach, see e.g. the methods reviewed in [35, 36], which covers different modeling formalisms used in gene regulatory network inference (GRN). The use of these techniques is not limited to GRNs; they can be applied to cellular networks in general [37]. Detailed comparisons of some of these methods can be found in several studies [4, 38–40]. Some state-of-the-art information-theoretic methods for network inference are ARACNe [41], and its extensions TD-ARACNE [42] and hARACNe [43], Context Likelihood of Relatedness, (CLR) [44], Minimum Redundancy Networks (MRNET) [45], three-way Mutual Information (MI3) [46], and Mutual Information Distance and Entropy Reduction (MIDER) [47], to name a few. All of them are based on estimating some information-theoretic quantity from the data and applying some criterion for determining the existence of links between pairs of variables. While the details vary from one method to another, it is difficult to single out a clearly “best” method. Instead, it has become clear in recent years that every method has its weaknesses and strengths, and their performance is highly problem-dependent; hence, the best option is often to apply “wisdom of crowds” methods, akin to the ensemble approach described above, as suggested by the results of recent DREAM challenges [48, 49]. In this spirit, recent software tools aim at facilitating the combined use of several methods [50].

Here, we present SELDOM (enSEmbLe of Dynamic lOgic-based Models), a method developed with the double goal of inferring network topologies, *i.e.* finding the set of causal interactions between a number of biological entities, and of generating high quality predictions about the behaviour of the system under untested experimental perturbations (also known as out-of-sample cross-validation). SELDOM makes no *a priori* assumptions about the model structure, and hence follows a completely data-driven approach to infer networks using mutual information. At the core of SELDOM is the assumption that the information contained in the available data will not be enough to successfully reconstruct a unique network. Instead, it will be generally possible to find many models that provide a reasonable description of the data, each having its own individual bias. Hence SELDOM infers a number of plausible networks, and uses them to generate an ensemble of logic-based dynamic models, which are trained with experimental data and undergo a model reduction procedure in order to mitigate overfitting. Finally, the simulations of the different models are combined into a single ensemble prediction, which is better than the ones produced by individual models.

The remainder of this paper is organised as follows. First, the Methods section provides a step by step description of the procedure followed by SELDOM. Then a number of experimental and *in silico* case studies of signaling pathways of different sizes and complexity are presented. In the Results section the performance of SELDOM is tested on these case studies and benchmarked against other methods. We finish by presenting some conclusions and guidelines for future work.

## Methods

**Fig 1:**
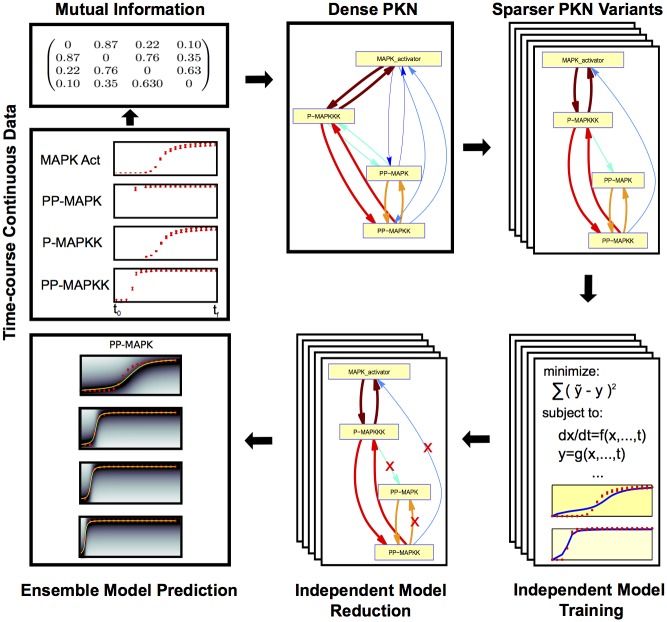
SELDOM workflow. The experimental data is used to build an adjacency (a dense DDN) matrix based on the mutual information of all pairs of variables. Through a simple sampling scheme, and limiting the maximum in-degree for each node, a set of more sparse DDNs are generated. Each individual DDN is then used as a scaffold for independent model training and model reduction problems. The resulting models are used to form an ensemble which is able to produce predictions for state trajectories under untested experimental conditions.

The SELDOM workflow, outlined in Fig 1, combines elements from information theory, ensemble modeling, parametric dynamic model identification, logic-based modeling and model reduction. The final objective is to provide high quality predictions of dynamic behavior even for untested experimental conditions. The method starts from time-course continuous experimental data ($\tilde{y}$) and uses data-driven networks (DDNs) as intermediate scaffolds. The workflow can be roughly divided into the following 5 steps:

1. Dense DDN inference using mutual information (MI) from experimental data $\tilde{y}$: build an adjacency (dense DDN) matrix based on the mutual information of all pairs of measured variables.
2. Sampling of DDNs: sample $n_{ℳ}$ data-driven networks (DDNs) based on the MI.
3. Independent model training: parametric identification of a set of ordinary differential equation models based on the DDNs.
4. Independent model reduction: iterative model reduction procedure of the individual models via a greedy heuristic.
5. Ensemble prediction: build ensemble of models to obtain predictions for state trajectories under untested experimental conditions.

The term network topology is defined here as a directed graph *G*. A directed graph (digraph) is a graph where all the edges are directed. The term node or vertex refers to a biological entity such as a protein, protein activity, gene, etc. A directed edge (interaction) starting from node *v*_*i*_ and pointing to *v*_*j*_ implies that the behavior of node *v*_*i*_ interferes with the behavior of node *v*_*j*_. In this case, *v*_*i*_ is said to be adjacent to *v*_*j*_. The in-degree of node *v*_*i*_ (*deg*^−^(*v*_*i*_)) is the number of edges pointing to *v*_*i*_. The directed graph *G* is composed of the ordered pair *G*(*V*(*G*), *E*(*G*)), where *V*(*G*) is the set of *n* vertices and *E*(*G*) is the set of *m* edges.

The input to the SELDOM algorithm is an experimental data-set formatted as a MIDAS file [51] and the maximum in-degree (*deg*^−^(*v*_*i*_)) allowed for each node in the networks sampled. The MIDAS file should specify for each experiment the observed signals, the observation times and the treatments/perturbations applied. Two types of perturbations are currently supported: inhibitors and stimuli. These are typical in most experimental studies of signaling pathways, where inhibitors are e.g. small molecules blocking kinase function, and stimuli are upstream ligands (e.g. hormones) whose initial concentration can be manipulated.

### Mutual information

The mutual information $MI({\tilde{y}}_{i},{\tilde{y}}_{j})$ between two random variables ${\tilde{y}}_{i}$ and ${\tilde{y}}_{j}$ is a measure of the amount of information that one random variable contains about another. It can also be considered as the reduction in the uncertainty of one variable due to the knowledge of another. It is defined as follows:

$$\text{MI}\left({\tilde{y}}_{i},{\tilde{y}}_{j}\right)=\sum_{\epsilon =1}^{n_{\epsilon }}\sum_{s=1}^{n_{s}^{\epsilon }}p\left({\tilde{y}}_{i}^{\epsilon ,s},{\tilde{y}}_{j}^{\epsilon ,s}\right)log\;\frac{p({\tilde{y}}_{i}^{\epsilon ,s},{\tilde{y}}_{j}^{\epsilon ,s})}{p\left({\tilde{y}}_{i}^{\epsilon ,s}\right)p\left({\tilde{y}}_{j}^{\epsilon ,s}\right)}$$ (1)

where ${\tilde{y}}_{i}$ and ${\tilde{y}}_{j}$ are discrete random vectors with probability mass functions *p*(*x*) and *p*(*y*), and log is usually the logarithm to the base 2, although the natural logarithm may also be used.

Since mutual information is a general measure of dependency between variables, it can be used for inferring interaction networks: the stronger the interaction between two network nodes, the larger their mutual information. If the probability distributions $p({\tilde{y}}_{i})$ and $p({\tilde{y}}_{j})$ are known, $MI({\tilde{y}}_{i},{\tilde{y}}_{j})$ can be derived analytically. In network inference applications, however, this is not possible, so the mutual information must be estimated from data, a task for which several techniques have been developed [52]. In the present work we calculate mutual information using the empirical estimator included in the R package *minet* [53].

### Sampling data-driven networks

Whatever the approach used to estimate the MI, estimation leads to errors, due to factors such as limited measurements or noisy data. Therefore, it is often the case that MI is over-estimated, which results in false positives. Network inference methods usually adopt strategies to detect and discard false positives. For example, ARACNe uses the data processing inequality, which states that, for interactions of the type *X* → *Y* → *Z*, it always holds that MI(*X*, *Y*) ≥ MI(*X*, *Z*). Thus, by removing the edge with the smallest value of a triplet, ARACNe avoids inferring spurious interactions such as *X* → *Z*. However, this in turn may lead to false negatives.

In the present work we are interested in building DDNs that are as dense as possible, in the sense that these should ideally contain all the real interactions, which leads to containing some false positives too (the issue of the false positives will be handled in the independent model reduction step). However, the subsequent dynamic optimization formulation used to train the models benefits from limiting the number of interactions (i.e. the number of decision variables grows very rapidly with the in-degree).

To find each DDN, we build an adjacency matrix using the array $MI({\tilde{y}}_{i},{\tilde{y}}_{j})$. Each column *j* represents the edges starting from *v*_*i*_ and pointing to *v*_*j*_. From this vector we iteratively select as many edges as the maximum in-degree (a pre-defined parameter of the method). In each selection step, an edge is chosen with a probability proportional to $MI({\tilde{y}}_{i},{\tilde{y}}_{j})$. This process is repeated for every node.

### From interaction networks to dynamic models

The DDNs obtained in the previous step represent a set of possible directed interactions. To characterize the dynamics of these interactions we use the multivariate polynomial interpolation technique [54, 55], which transforms discrete models into continuous ones described by ordinary differential equations (ODEs). This interpolation is particularly well suited to represent signaling pathways, since it is able to describe a wide range of behaviours including combinatorial interactions (OR, AND, XOR, etc). Having an ODE-based description allows us to simulate the time courses of the model outputs. By minimizing the difference between those predictions and the experimental data we can obtain a trained model. In the following lines we present the mathematical formulation of the dynamic equations, and in the next subsection we describe the model calibration approach.

Let us represent a Boolean variable by *x*_*i*_ ∈ {0, 1}. In logic-based ODE models each state variable ${\bar{x}}_{i}$ is a generalization of a Boolean variable, and can have continuous values between zero and one, that is, ${\bar{x}}_{i}\in [0\;1]$. Every *i*^*th*^ state variable in the model, ${\bar{x}}_{i}$, represents a species whose behaviour is governed by a set *ϕ*_*i*_ of *N*_*i*_ variables which act as upstream regulators (${\bar{x}}_{i1},\ldots ,{\bar{x}}_{iN_{i}}$). Each of the *N*_*i*_ regulators in *ϕ*_*i*_ is listed by an index *ϕ*_*ik*_. For example, if a variable ${\bar{x}}_{5}$ is regulated by the following subset of *N*_5_ = 3 nodes: $\phi_{5}=\{{\bar{x}}_{3},{\bar{x}}_{7},{\bar{x}}_{8}\}$, we would have three indices *ϕ*_5 1_ = 3, *ϕ*_5 2_ = 7, and *ϕ*_5 3_ = 8.

For each interaction we describe the nonlinearity that governs the relation between the upstream regulators ${\bar{x}}_{k}$ and the downstream variable ${\bar{x}}_{i}$ using the normalized Hill function $H_{\phi_{i_{k}}}$. For each index *ϕ*_*ik*_ the normalized Hill function has the form:

$$H_{\phi_{ik}}=\frac{{\bar{x}}_{\phi_{ik}}^{n_{\phi_{ik}}}}{{\bar{x}}_{\phi_{ik}}^{n_{\phi_{ik}}}+k_{\phi_{ik}}^{n_{\phi_{ik}}}}\cdot \left(1+k_{\phi_{ik}}^{n_{\phi_{ik}}}\right)$$ (2)

We have chosen the normalized Hill function because it is able to represent the switch-like behaviour seen in many molecular interactions [54], as well as other simpler behaviours such as Michaelis Menten type kinetics. The shape of this curve is defined by the parameters $n_{\phi_{i_{k}}}$ and $k_{\phi_{i_{k}}}$.

Using these Hill functions we write the continuous homologue of the Boolean update function for variable ${\bar{x}}_{i}$ as:

$$\bar{B_{i}}=\sum_{x_{i1}=0}^{1}\ldots \sum_{x_{iN_{i}}=0}^{1}\left[w_{x_{i1},\ldots ,x_{iN_{i}}}\cdot \prod_{k=1}^{N_{i}}\left(x_{ik}H_{\phi_{ik}}+\left[1-x_{ik}\right]\left[1-H_{\phi_{ik}}\right]\right)\right]$$ (3)

where the w* are parameters that define the model structure. Note that each w* has *N*_*i*_ subindices, that is, as many subindices as regulators (this number varies from one variable to another). We also remark that *x*_*i*1_, …, *x*_*iNi*_ are Boolean variables, so the term $\sum_{x_{i1}=0}^{1}\ldots \sum_{x_{iN_{i}}=0}^{1}[\ldots ]$ represents sums where the *x*_i_* elements have values zero or one.

The time evolution of ${\bar{x}}_{i}$ is then given by:

$$\dot{\bar{x_{i}}}=\left(\bar{B_{i}}-{\bar{x}}_{i}\right)\cdot \frac{1}{\tau_{i}}$$ (4)

where *τ*_*i*_ can be seen as the lifetime of species *x*_*i*_.

This representation can reproduce several behaviours of interest (see Table 1). For example, if we consider that a variable is controlled by two regulators, an AND type behaviour would be defined by setting *w*_*i*,1,1_ to 1 and the other *w*’s (*w*_*i*,0,0_, *w*_*i*,0,1_, and *w*_*i*,1,0_) to 0. On the other hand, the OR gate can be represented by setting *w*_*i*,1,0_ and *w*_*i*,0,1_ to 1, and *w*_*i*,1,1_ and *w*_*i*,0,0_ to 0. By linear combinations of these terms it is possible to obtain any of the 16 gates that can be composed of two inputs.

**Table 1:** Table illustrating the multivariate polynomial interpolation function and model reduction.

*x*_1_	*x*_2_	${\bar{B}}_{i}=\ldots$	${\bar{B}}_{i}^{*}=\ldots$
0	0	$w_{i,0,0}\cdot \left(1-H_{\phi_{i1}}\right)\cdot \left(1-H_{\phi_{i2}}\right)+\ldots .$	$w_{i,0,0}\cdot \left[1-H_{\phi_{i1}}\right]\cdot 1+\ldots$
0	1	$w_{i,0,1}\cdot \left(1-H_{\phi_{i1}}\right)\cdot H_{\phi_{i2}}+\ldots .$	0 + …
1	0	$w_{i,1,0}\cdot H_{\phi_{i1}}\cdot \left(1-H_{\phi_{i2}}\right)+\ldots .$	$w_{i,1,0}\cdot H_{\phi_{i1}}\cdot 1+\ldots$
1	1	$w_{i,1,1}\cdot H_{\phi_{i1}}\cdot H_{\phi_{i2}}$	0

The multivariate polynomial interpolation function, ${\bar{B}}_{i}$, is simplified by setting $H_{\phi_{\text{ij}}}$ to zero, resulting in function ${\bar{B}}_{i}^{*}$. In practice this is equivalent to removing the edge *e*_*ij*_. In the model reduction procedure, the remaining parameters are then estimated starting from the best known solution, and the new (simpler) model is accepted if it is better according to the Akaike information criterion.

This framework is very general and requires very few assumptions about the system under study. This comes at the cost of a large number of parameters to estimate: in principle, both Hill constants ($n_{\phi_{i_{k}}}$ and $k_{\phi_{i_{k}}}$) are unknown, as well as the structure parameters w* and the lifetime parameter *τ*_*i*_. The following subsection provides a formal definition of the parameter estimation problem.

### Independent model training

For each DDN it is possible to obtain the corresponding dynamic model automatically, as explained in the preceding subsection. Every such model has a number of unknown parameters: for each variable, one or several n*, k*, w*, and τ* have to be estimated. To this end we formulate a parameter estimation problem in which the objective function (*F*) is the squared difference between the model predictions (*y*) and the experimental data ($\tilde{y}$). The goal is to minimize this cost function for every experiment (*ϵ*), observed species (*o*) and sampling point (*s*). The model prediction obtained by simulation (*y*) is a discrete data set given by an observation function (*g*) of the model dynamics at time *t*. This parameter estimation problem is formally defined as:

$$\begin{array}{c} \begin{array}{cccc} & \underset{n_{*},k_{*},\tau_{*},w_{*}}{\text{minimize}} & & F=\sum_{\epsilon =1}^{n_{\epsilon }}\sum_{o=1}^{n_{o}^{\epsilon }}\sum_{s=1}^{n_{s}^{\epsilon ,o}}{\left({\tilde{y}}_{s}^{\epsilon ,o}-y_{s}^{\epsilon ,o}\right)}^{2}\\ & \text{subject}\;\text{to}\\ & & & N_{i}=deg^{-}\left(v_{i}\right)\\ & & & \phi_{i}=\left\{j\;|e_{ij}=1\right\},\;i=1,\ldots ,n,\;j=1,\ldots ,n\\ & & & H_{\phi_{ik}}=\frac{{\bar{x}}_{\phi_{ik}}^{n_{\phi_{ik}}}}{{\bar{x}}_{\phi_{ik}}^{n_{\phi_{ik}}}+k_{\phi_{ik}}^{n_{\phi_{ik}}}}\cdot \left(1+k_{\phi_{ik}}^{n_{\phi_{ik}}}\right)\\ & & & \bar{B_{i}}=\sum_{x_{i1}=0}^{1}\ldots \sum_{x_{iN_{i}}=0}^{1}\left[w_{x_{i1},\ldots ,x_{iN_{i}}}\cdot \prod_{k=1}^{N_{i}}\left(x_{ik}H_{\phi_{ik}}+\left[1-x_{ik}\right]\left[1-H_{\phi_{ik}}\right]\right)\right]\\ & & & \dot{\bar{x_{i}}}=\left(\bar{B_{i}}-{\bar{x}}_{i}\right)\cdot \frac{1}{\tau_{i}}\\ & & & {\bar{x}}_{i}\left(t_{0}\right)={\bar{x}}_{i0}\\ & & & y=g(\bar{x})\\ & & & 0\leq w_{i}\leq 1\\ & & & {\text{LB}}_{n}\leq n\leq {\text{UB}}_{n}\\ & & & {\text{LB}}_{k}\leq k\leq {\text{UB}}_{k}\\ & & & {\text{LB}}_{\tau }\leq \tau \leq {\text{UB}}_{\tau }, \end{array} \end{array}$$ (5)

where *g* is the output function, which often consists of a subset of the states (if all the states are measured, it is simply $y=g(\bar{x})=\bar{x}$).

The upper and lower bounds of the parameters (e.g. *LB*_*k*_) are set to values as wide as possible based on their biochemical meaning and prior knowledge, if existing.

We have recently shown [56] how to train a more constrained version of this problem using mixed-integer nonlinear programming (MINLP). Here, due to its size, the problem is first relaxed into a nonlinear programming (NLP) problem. The corresponding parameter estimation problem is non-convex, so we use the scatter search global optimization method [57] as implemented in the MEIGO toolbox [58].

We note that performing parameter estimation entails repeatedly solving an initial value problem (IVP), which consists of integrating the ODEs from a given initial condition in order to obtain the time course simulation of the model output *y*. Several studies that have considered simultaneous network inference and parameter estimation have chosen discretization methods for the solution of the IVP [7, 8]. This has some advantages regarding computational tractability, but forces the $\dot{x}$ values to be estimated directly from noisy measurements, which is especially challenging when samples are sparse in time. Instead, here we solve the IVP using a state-of-the-art solver for numerical integration of differential equations, CVODE, which is included in the SUNDIALS package [59].

### Independent model reduction

Model reduction is a critical step in SELDOM due to two reasons: (i) we are interested in reducing the network to keep only interactions that are strictly necessary to explain the data (feature selection); (ii) following Occam’s razor principle, it is expected that the ideal model in terms of generalization is the one with just the right level of complexity [60].

Our model reduction procedure is partially inspired by the work of Sunnaker et al [61], where a search tree starting from the most complex model is used to find the complete set of all the simplest models by iteratively deleting parameters. In contrast to [61], we implement a greedy heuristic algorithm. While this method does not offer guarantees of finding the simplest model, it drastically reduces the computational time needed to find the simplest solution. This heuristic helps to maintain diversity in the solutions and ensures that spurious edges are not considered. The iterative model reduction procedure is described in Algorithm 1. At each step (i.e. for each edge), the constraint $H_{\phi_{i_{k}}}$ is set to zero (see Table 1) and the model is calibrated with a local optimization method, Dynamic Hill Climbing (DHC) [62]. To avoid potential bias caused by the model structure, edges are deleted in a random order.

To decide about the new simplified model we use the Akaike information criterion (AIC), which for the purpose of model comparison is defined as:

$$AIC=2K+2n\cdot ln\left(\frac{F}{n}\right),$$ (6)

where *K* is the number of active parameters. The theoretical foundations for this simplified version of the AIC can be found in [63].

**Algorithm 1:** Greedy heuristic used to reduce the model. At each step of the model reduction the new (simpler) solution is tested against the previous (more complex) one using the Akaike information criteria (AIC).

**Data:** Time-course continuous data $\tilde{y}$, a graph *G*_*a*_(*V*, *E*) and the optimal parameters (*n*, *k*, *τ*, *w*)

**Result:** A simplified graph *G*_*a*_(*V*, *E*)*

**for** *each* $e_{\phi_{i_{k}}}$ ∈ *G*_*a*_ **do**

 $\underset{n_{*},k_{*},\tau_{*},w_{*}}{\text{minimize}}\;F=\sum_{\epsilon =1}^{n_{\epsilon }}\sum_{o=1}^{n_{o}^{\epsilon }}\sum_{s=1}^{n_{s}^{\epsilon ,o}}{\left({\tilde{y}}_{s}^{\epsilon ,o}-y_{s}^{\epsilon ,o}\right)}^{2}$

 subject to

 $H_{\phi_{i_{k}}}$ = 0

 …

 **if** AIC(n*, k*, τ*, w*) < AIC(*n*, *k*, *τ*, *w*) **then**

  *E*_*a*_ ← *E*_*a*_\$e_{\phi_{i_{k}}}$

  {*n*, *k*, *τ*, *w*}←{n*, k*, τ*, w*}

 **end**

**end**

### Ensemble model prediction

To generate ensemble predictions for the trajectories of state *x*_*i*_, SELDOM uses the median value of *x*_*i*_ across all models for a given experiment *i*_*exp*_ and sampling time *t*_*s*_. This is the simplest way to combine a multi-model ensemble projection. More elaborate schemes for optimally combining individual model outputs exist. Gneiting et al. [64] point out that such statistical tools should be used to obtain the full potential of a multi-model ensemble. However, the selection of such weights requires a metric describing the model performance under novel untested conditions (*i.e.* forecasting), and finding such metric is a non trivial task. For example, in the context of weather forecasting, Tebaldi et al [27] point out that, in the absence of a metric to quantify model performance for future projections, the usage of simple average is a valid and widely used option that is likely to improve best guess projections due to error cancellation from different models.

### Implementation

SELDOM has been implemented mainly as an R package (R version 2.15), with calls to solvers implemented in C/C++ or Fortran codes using Intel compilers. The SELDOM code is open source and it is distributed as is (with minimal documentation), along with the scripts needed to reproduce all the results and figures.

SELDOM can be installed and run in large heterogeneous clusters and supercomputers. This configuration allows to reduce computation times thanks to the adoption of several parallelization strategies. Model training and reduction are embarrassingly parallel tasks (i.e. they can be performed independently for each individual model). They are automated using shell scripts and a standard queue management system. In addition to this parallelization layer (at the level of individual model training and reduction) there is another parallelization layer at a lower level: for each model, each experiment is simulated in a parallel individual thread using openMP [65], which allows exploiting multi-core processors.

The dynamic optimization problem associated to model training is solved as a master (outer) nonlinear programming problem (NLP) with an inner initial value problem (IVP). The NLPs are solved using the R package MEIGOR [58], with the evaluation of the objective function performed in C code. The solutions of IVPs are obtained by using the CVODE solver [59].

The experimental data is provided using the MIDAS file format, and it is imported and managed using CellNOptR [66].

### Case studies

To assess the performance of SELDOM, we have chosen a number of *in silico* and experimental problems in the reconstruction of signaling networks. Table 2 shows a compact description of some basic properties of these case studies along with a more convenient short name for the purpose of result reporting.

**Table 2:** An overview of the characteristics of all case studies approached in this work.

case study	Short name	Reference	Data	*N*_*obs*_	*N* Train	*N* Prediction	*deg*^−^(*v*_*i*_)
1a	MAPKp	[67]	*in silico*	4	10	10	*A* = 1, *B* = 2, *C* = 3
1b	MAPKf	[67]	*in silico*	13	10	10	*A* = 3, *B* = 4, *C* = 5
2	SSP	[68]	*in silico*	13	10	36	*A* = 3, *B* = 4, *C* = 5
3	DREAMiS	[69]	*in silico*	2	20	128	*A* = 3, *B* = 4, *C* = 5
4a	DREAMBT20	[70]	Experimental	54	29	8	*A* = 3, *B* = 4, *C* = 5
4b	DREAMBT549	[70]	Experimental	52	24	8	*A* = 3, *B* = 4, *C* = 5
4c	DREAMMCF7	[70]	Experimental	47	32	8	*A* = 3, *B* = 4, *C* = 5
4d	DREAMUACC812	[70]	Experimental	52	32	8	*A* = 3, *B* = 4, *C* = 5

The most relevant factors are the number of observed variables, the number of experiments considered for training, the number of experiments considered for prediction and the different maximum in-degrees tested in each case study.

For each case study, two data-sets were derived, one for inference and the second one for performance analysis. We highlight that training and performance assessment data-sets are not just two realizations of the same experimental designs; they were obtained by applying different perturbations, such as different initial conditions or the introduction of inhibitors either experimentally or *in silico*.

The logic-based ODE framework is only able to simulate values between 0 and 1. Therefore,in each case-study, we have normalized the data by dividing every value of a measured protein by the maximum value of that protein found across all conditions and time points in the training data-set. This normalization procedure also facilitates comparison across case-studies.

#### Case studies 1a and 1b: MAPK signaling pathway

Huang et al. [67] developed a model explaining the particular structure of the mitogen-activated protein kinases (MAPKs). This is a highly conserved motif that appears in several signaling cascades (ERK, p38, JNK) [71] composed of 3 kinases. Essentially, Huang et al [67] explain how this arrangement of three kinases sequentially phosphorylated in different sites allows that a graded stimuli is relayed in a ultrasensitive switch-like manner.

**Fig 2:**
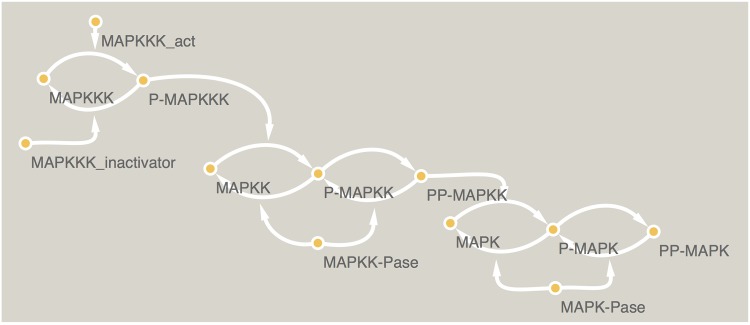
MAPK signaling network. The model by Huang et al. [67] was used to generate pseudo-experimental data for two sub-problems. The first (MAPKp) partially observed (MAPK-PP, MAPKK-PP and MAPKKK), and the second fully observed MAPKf.

To create this benchmark, the model shown in Fig 2 was used to generate artificial data with no noise. The full system is composed of 12 ODEs. Based in this system, we have derived two case studies, one fully observed (MAPKf) and the second partially observed (MAPKp). The fully observed system is essentially the same as used in [47], while in the partially observed case only one phosporylation state per kinase was considered (MAPK-PP, MAPKK-PP and MAPKKK).

We highlight that the model representation used in SELDOM is particularly suitable to represent such compact descriptions of signaling mechanisms due to the usage of Hill functions. Additionally, looking at partially observed systems is well in line with experimental practice as state-of-the-art methods for studying signaling pathways are typically targeted to particular states (e.g. phosphorylation) of the proteins (e.g. kinases) involved in the signaling pathways.

Both the data-sets used for training and predictions are composed of 10 different experiments, each with different initial conditions and without added noise. The data used for MAPKp case study is a sub-set of the MAPKf data-set.

#### Case study 2: A synthetic signaling pathway

Resorting to logic-based ODEs, MacNamara et al [68] derived a synthetic model representative of a typical signaling pathway. The goal was to illustrate the benefits and limitations of different simulations for signaling pathways. This model includes three MAPK systems (p38, ERK and JNK1) and two upstream ligand receptors for EGF and TNF*α*. Apart from different on/off combinations of EGF and TNF*α*, the model simulations can be perturbed by inhibiting PI3K and RAF.

The training data-set is composed of 10 experiments with different combinations of ligands (EGF and TNF*α* on and off) and the inhibitors for RAF and PI3K.

The data-set used to assess performance was generated using the synthetic signaling pathway (SSP) model with the same combinations of EGF and TNF*α*, but changing the inhibitors. Instead of inhibiting PI3K and RAF, we generate new experiments by considering all other states observed with exception of EGF and TNF*α*. The final outcome is a validation data-set with 36 experiments.

Both data-sets (training and validation) were partially observed (11 out of 26 variables) and Gaussian noise (with standard deviation *σ* = 0.05 and 0 mean) was added. In this case study the inhibitors are implemented as:

$${\dot{\bar{x}}}_{\text{inh},i}=\left(\bar{B_{i}}-{\bar{x}}_{i}\right)\cdot \frac{1}{\tau_{i}}\cdot \left(1-{\text{inh}}_{i}\right),$$ (7)

where *inh*_*i*_ is chosen as 0.9.

#### Case study 3: HPN-DREAM breast cancer network inference, *in silico* sub-challenge

This is an *in silico* problem developed by the HPN-DREAM consortium. It is a synthetic problem that replicated the reverse phase protein array (RPPA) experimental technique for studying signalling pathways with multiple perturbations as realistically as possible. These perturbations often consist in manipulating ligand concentrations and adding small molecule inhibitors. To achieve this, the authors extended the model from Chen et al. [69], a large dynamic model of ErbB signaling pathways. The model was partially observed (17 variables) and perturbed with a noise model aimed at reproducing the RRPA experimental technique as accurately as possible. In addition to these 17 variables, 3 dummy variables consisting of noise were included to make the challenge even more difficult. All names in the model were replaced by aliases (eg. AB1, AB2, etc).

The training data-set is composed of 20 experiments obtained by considering different combinations of 2 ligands (off, low and high) and 2 small molecule inhibitors. The data-set used for performance assessment is composed of 128 experiments considering the inhibition of the other 15 observed states not considered in the generation of the training set and different combinations of ligand concentrations (off, low and high).

Regarding the implementation of the inhibitors, we followed the same strategy used in the SSP case-study where these are implemented under the assumption that an inhibitor inh_*i*_ of state *x*_*i*_ directly affects the concentration of *x*_*i*_. Such an assumption is based on the challenge design and made following the instructions of the challenge developers.

#### Case studies 4a to 4d: HPN-DREAM breast cancer network inference

One of the richest data-sets of this type was recently made publicly available in the context of the DREAM challenges (www.dreamchallenges.org). DREAM challenges provide a forum to crowdsource fundamental problems in systems biology and medicine, such as the inference of signaling networks [48, 70], in the form of collaborative competitions. This data-set comprised time-series acquired under eight extracellular stimuli, under four different kinase inhibitors and a control, in four breast cancer cell lines [70].

The HPN-DREAM breast cancer was composed of two sub-challenges. In the experimental sub-challenge the participants were asked to make predictions for 44 observed phosphoproteins, although the complete data-set was larger. As opposed to the *in silico* sub-challenge, the participants were encouraged to use all the prior knowledge they could use and the experimental protocol along with the real names of the measured quantities, used reagents, inhibitors, etc.

Using different combinations of inhibitors and ligands (on and off), the authors have generated a data-set for several cell-lines. An additional data-set generated with the help of a fourth inhibitor was kept unknown to the participants, who were asked to deliver predictions for several possible inhibitors.

Here, it is assumed that the inhibitors affect mostly the downstream activity of a given kinase. However, it is unknown how it actually influences the kinase concentration or the ability to measure it experimentally. Therefore, the mutual information matrix used to find DDN variants is computed here as:

$${\text{MIM}}_{\text{inh}}=\text{max}\left(\text{MIM}\left(\tilde{y}\cdot \left(1-{\text{inh}}_{i}\right)\right),\text{MIM}\left(\tilde{y}\right)\right)$$ (8)

where inh_*i*_ is a vector of the same size as $\tilde{y}$, filled with 0.9 when the inhibition is applied and with 0 otherwise. Regarding the implementation of the dynamic behaviour, this is performed by modifying $H_{\text{inh},\phi_{ik}}$ of an inhibited species *x*_*k*_ to:

$$H_{\text{inh},\phi_{ik}}=\frac{{\bar{x}}^{n_{\phi_{ik}}}\cdot \left(1-{\text{inh}}_{k}\right)}{{\bar{x}}^{n_{\phi_{ik}}}\cdot \left(1-{\text{inh}}_{k}\right)+k^{n_{\phi_{ik}}}}\cdot \left(1+k^{n_{\phi_{ik}}}\right)$$ (9)

## Results

### Numerical experiments and method benchmarking

In this section, we describe the numerical experiments carried to show the validity of our ensemble based approach. Besides particular considerations in the data preprocessing or additional constraints added to the dynamic optimization problem which depend on the prior knowledge existent about the case study at hand, SELDOM has two tuning parameters: the ensemble size and the maximum in-degree allowed in the training process. Thus, besides showing how the method performs and illustrating the process we also wanted to show that the method is relatively robust to the choice of these parameters and provide guidelines for the choice of such parameters in future applications.

For each case study we have chosen 3 in-degrees (A, B and C) which are shown in Table 2 and we have chosen a fairly large ensemble size of 100 models.

To assess performance in terms of training and predictive skills of the model, we use the root mean square error (RMSE):

$$\text{RMSE}=\sqrt{\frac{\sum_{\epsilon =1}^{n_{\epsilon }}\sum_{o=1}^{n_{o}^{\epsilon }}\sum_{s=1}^{n_{s}^{\epsilon ,o}}{\left[{\tilde{y}}_{s}^{\epsilon ,o}-y_{s}^{\epsilon ,o}\right]}^{2}}{\sum_{\epsilon =1}^{n_{\epsilon }}\sum_{o=1}^{n_{o}^{\epsilon }}n_{s}^{\epsilon ,o}}}$$ (10)

To assess performance in terms of network topology inference, we have chosen the area under precision recall (AUPR) curve, where precision (P) and recall are defined as (R):

$$P=\frac{\text{TP}}{\text{TP}+\text{FP}}$$ (11)

and

$$R=\frac{\text{TP}}{\text{TP}+\text{FN}},$$ (12)

where TP and FP correspond to the number of true and false positives, respectively and FN corresponds to the number of false negatives.

Other valid metrics exist, such as the Area Under the Receiver Operator Characteristic curve (AUROC). The ROC plots the recall, R, as a function of the false positive rate, FPR, which is defined as

$$\text{FPR}=\frac{\text{FP}}{\text{FP}+\text{TN}}.$$ (13)

However, it has been argued that ROC curves can paint an excessively optimistic picture of an algorithm’s performance [72], because a method can have low precision (i.e. large FP/TP ratio) and still output a seemingly good ROC. Hence we have chosen to use the AUPR measure instead.

### Predicting trajectories for new experimental perturbations

**Fig 3:**
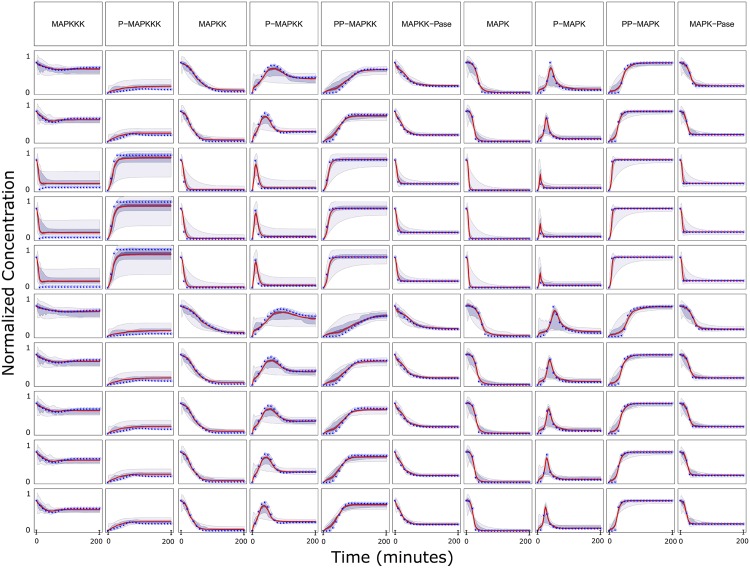
Time course predictions for case study 1b (MAPKf). The median in red is surrounded by the predicted non-symmetric 20%,60% and 95% confidence intervals.

**Fig 4:**
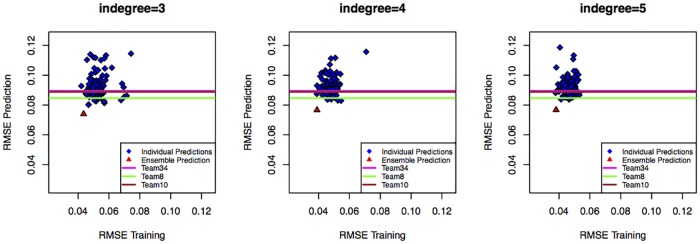
Relationship between training and prediction RMSE for case study 3 (DREAMiS). The prediction RMSE is plotted here against the training RMSE for each individual model (blue) and the ensemble (red). RMSE scores for the top 3 performing teams in the HPN-DREAM *in silico* time-course prediction sub-challenge (Team34, Team8 and Team10) are also shown as colored lines.

The training data-sets in each case-study were used to obtain time-course trajectories for untested conditions. The type of trajectories obtained with SELDOM is illustrated with the help of Fig 3 which shows the time-course predictions for different conditions in the case-study 1b (MAPKf). Ensemble trajectories for training and untested conditions are also given for other case-studies in S1 Text and S2 Text, respectively. In most cases the ensemble behaved better than the model with lowest RMSE training value. This effect is particularly evident in the DREAMiS case-study and is reflected in Fig 4. Similar plots are also given for other case studies in S3 Text. In a number of case-studies (DREAMiS, DREAMBT20, DREAMBT549, DREAMMCF7 and DREAMUACC812) there is little correlation between the training RMSE and the prediction RMSE.

**Fig 5:**
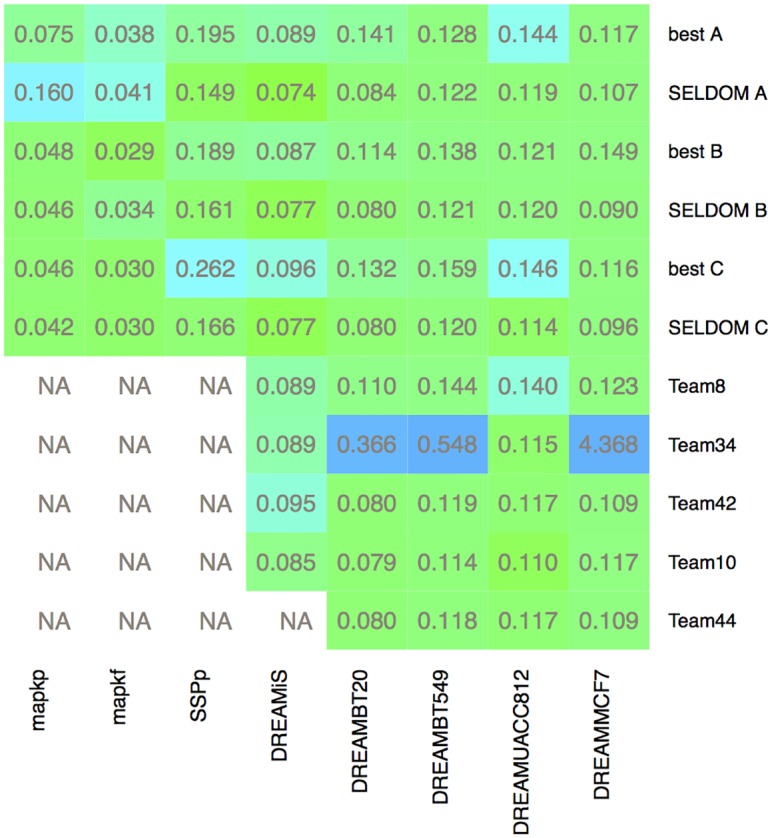
Heatmap with RMSE scores for different methods and case studies. The color scheme represents RMSE scores normalized by case-study in order to emphasize differences between methods. The color scale moves from green (low RMSE) to blue (high RMSE). The numeric values of the RMSE scores for each method/case-study are also provided in each corresponding cell. SELDOM B and SELDOM C were clearly the most robust strategies doing very well in all problems.

In Fig 5, we show the overall picture regarding the predictive skills. Two strategies were considered for the generation of predictions: the best individual model and SELDOM. The RMSE values were normalized by problem and plotted as an heatmap. Additionally, for DREAMiS, DREAMBT20, DREAMBT549, DREAMMCF7 and DREAMUACC812 we added the prediction RMSE values for the top 3 performing participants in the corresponding DREAM sub-challenges (experimental and *in silico*). To compute these RMSE scores we downloaded the participants predictions (available online) and normalized the data by using the maximum value of each measured signal found in the experimental data-set. The greatest gain of using an ensemble approach as shown here is in robustness. The effect of the model reduction was relatively small (yet not neglectable) in terms of RMSE for prediction.

Comparing SELDOM results with those generated during the DREAM challenge, we managed to generate predictions with lower RMSE score than the top 3 participants (Team34, Team8 and Team10) of the *in silico* time-course predictions sub-challenge. Team34 (ranked first) built consensus networks generated by different inference algorithms applied to multiple subsets of the data. To generate the time-course predictions the previously mentioned team used generalized linear models informed by the inferred networks [70]. Regarding the experimental sub-challenge for most cases we obtained similar results to those of the top 3 participants (Team44, Team42 and Team10) with the exception of cell-line DREAMMCF7 where results where slightly better.

**Fig 6:**
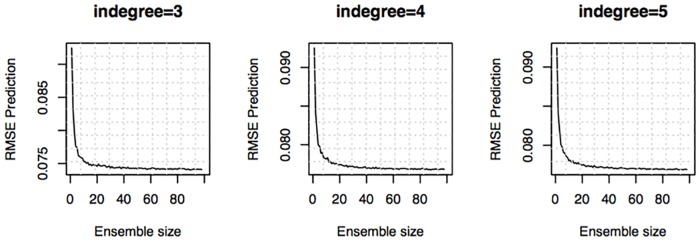
Ensemble predictive skill depending on ensemble size (case study DREAMiS). This curve was computed by bootstrapping multiple $n_{ℳ}$ models from the available 100 models, *i.e.* we sampled multiple realizations of the individual predictions for the same ensemble size and computed the average value. These curves converge asymptotically and show that the chosen ensemble size parameter is adequate. Equivalent predictions could have been obtained with smaller ensemble sizes.

The choice of ensemble size parameter affects the predictive skill of the ensemble and the computational resources needed to solve the problem. To verify if this choice was an appropriate one we plotted the average prediction RMSE as a function of the number of models $n_{ℳ}$ used to generate the ensemble. The average RMSE was computed by sampling multiple models from the family of models available to compute the trajectories. This is shown in Fig 6 for the DREAMiS case-study. Similar curves are given for all case studies as supporting information in S4 Text. With the exception of the combination MAPKp/SELDOM A the outcome for all case-studies is that SELDOM would have done similarly well with a smaller number of models and the prediction RMSE *versus* $n_{ℳ}$ always converged asymptotically. The mediocre results from the MAPKp/SELDOM A combination appear to be the result of a poor choice for the maximum in-degree parameter (A = 1) which is too small.

#### Ensemble for network inference

To assess the performance of SELDOM for the network topology inference problem, we compared SELDOM with a number of methods implemented in the *minet* package [53]: MRNET [45], MRNETB [73], CLR [44] and ARACNE [41]. This comparison is particularly pertinent in this case as the estimation of the mutual information is done using the same method and parameterization. However, these methods are not designed to recover directed networks. To surmount this limitation, we have introduced the comparison with two other methods for directed networks, TDARACNE [42] and MIDER [47].

**Fig 7:**
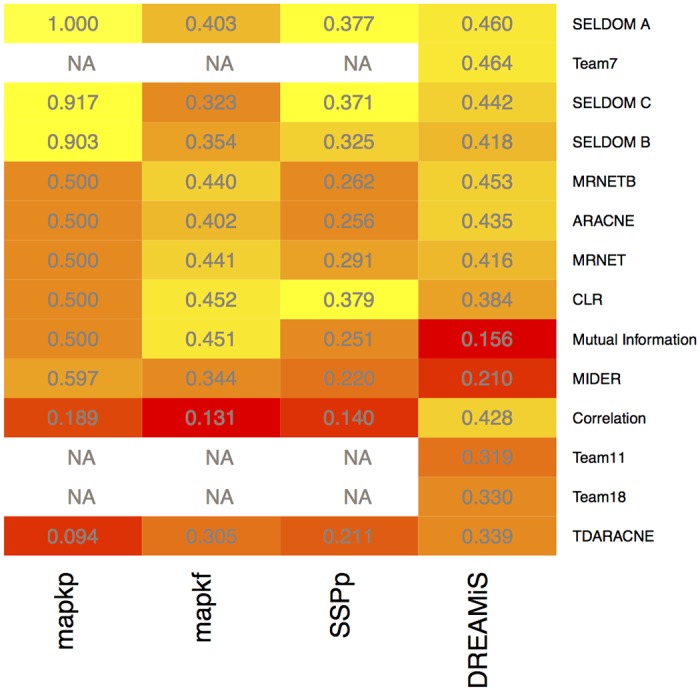
Heatmap with AUPR scores for different methods and case studies. The color scheme represents AUPR scores normalized by case-study in order to emphasize differences between methods. The color scale moves from red (low AUPR) to yellow (high AUPR). The numeric values of the AUPR scores for each method/case-study are also provided in each corresponding cell. The sparsest version of SELDOM (A) did consistently well in all the case studies. SELDOM B and C did an average job with MAPKf but provided good solutions for all other case-studies. The comparisons are only provided for *in silico* problems with known solution. Additionally, the solution for the top performing teams in the DREAM challenge is only available for DREAMiS.

In Fig 7, we show the overall results regarding the ability of SELDOM and other network inference methods to reverse engineer the known synthetic networks associated with the models used to generate the data. Comparing with static inference methods, SELDOM behaved consistently well in terms of providing networks with high AUPR score. The sparsest configuration of SELDOM (A) provided the most interesting results.

For case study 3 (DREAMiS), we compared the ensemble networks from SELDOM with the networks inferred by the top 3 participants of the *in silico* network inference sub-challenge (Team7, Team11 and Team18). To perform this comparison we downloaded the networks which are available online and computed the AUPR scores. These AUPR scores are shown in Fig 7. SELDOM found networks with AUPR scores that were marginally lower than the top performing participant (Team7) that used GLN [74], a method based on a novel functional *χ*^2^ test to examine functional dependencies between variables. On the other hand, SELDOM obtained AUPR scores higher than the participants ranked second (Team11) and third (Team18).

Without the independent model reduction step, the results were mediocre regarding the inference of the network topology. The independent model reduction is fundamental for the performance of SELDOM as a method for network inference and the information contained in the dynamics can help discard spurious links. This is illustrated in S5 Text where the mean AUPR score is shown for multiple bootstrapped realizations of the ensemble network with and without applying model reduction. Additionally S6 Text is provided as supporting information where the AUPR curves and AUROC scores are given for all the methods tested in the different case-studies.

We also considered how to validate SELDOM results using experimental data and its capabilities to generate new hypothesis. With this aim, we compared the inferred networks with the current knowledge, as detailed in S7 Text. Briefly, we started by selecting a subset of interactions consistently recovered by SELDOM across all cell-lines. We then searched the literature in a systematic manner to assess the agreement of these predictions with the knowledge currently available in manually curated databases. To this end we used the OmniPath software [75], a comprehensive compendium of literature-curated pathway resources. We found literature support for most (16 out of 20) of the interactions reported with high confidence in all cell-lines. This fact indicates that the predictions made by SELDOM are, generally, consistent with existing biological knowledge. Furthermore, in the cases in which we did not find in OmniPath reports of any interactions, we searched the literature manually and analysed each of the four cases individually. From this analysis we concluded that (i) one of the candidate interactions is a probable true positive (details in S7 Text), (ii) a second one has actually been reported in the literature as an indirect regulator, (iii) a third case is a probable false positive, and (iv) a last case which seems to be a good hypothetical candidate which would require further testing. For this last case we found no literature support for alternative mechanisms. This latter case is an illustrative example of using SELDOM to generate new hypotheses.

## Discussion

In this paper we have presented a novel method for the generation of dynamic predictions in signaling networks. The method (enSEmbLe of Dynamic lOgic-based Models, SELDOM) handles the indeterminacy of the problem by generating, in a data-driven way, an ensemble of dynamic models combining methods from information theory, global optimization and model reduction. It should be noted that although this method is data-driven (not requiring any prior knowledge of the network), it produces logic-based dynamic models which allow for mechanistic interpretations and are capable of making predictions in different conditions than those used for model calibration.

In this study we focused mostly on the methodological aspects of SELDOM and performed comparisons based on standard metrics. SELDOM can provide cell-specific networks that can be explored to formulate biological hypotheses and guide the design of new experiments to validate them (as an example, S7 Text shows the networks obtained with SELDOM for different cell-lines of the HPN-DREAM experimental data sub-challenge).

We applied SELDOM to a number of challenging experimental and *in silico* signal transduction case-studies, including the recent HPN-DREAM breast cancer challenge. Regarding network inference, the ensemble approach did systematically well in all of the *in silico* cases considered in this work. This suggests that exploiting the information contained in the dynamics, as SELDOM does, helps the network inference problem allowing to disregard spurious interactions. We have also shown that, unlike most network inference methods, SELDOM is also capable of making robust dynamic predictions in untested experimental conditions. For this task, SELDOM’s ensemble predictions were not only consistently better than the outcomes of individual models, but also often outperformed the state of the art represented by the best performers in the HPN-DREAM challenge. It should be noted that the use of mechanistic dynamic models provides great flexibility regarding the simulation of complex time-varying situations. For example, it is not only possible to simulate inhibitions of several nodes in order to determine which combination produces the desired response, but also to test the outcome of sequential interventions (i.e. taking place at different times during the course of a treatment), which would be impossible to model using statistical approaches that lack mechanistic detail. Another important application is the design of optimal dynamic experiments, i.e. those where the inputs acting as stimuli are designed as time-varying functions ([76, 77]). Furthermore, it is also possible to use the ensemble for extracting biological hypotheses about poorly known parts of a signaling pathway.

The proposed SELDOM pipeline is flexible and can be adapted to any signaling or gene regulation dataset obtained upon perturbation, even if prior knowledge is not available. At the same time, it is also able to incorporate prior knowledge about a problem, for instance in the form of constraints (e.g. the small-molecule inhibitors used in the HPN-DREAM challenge case studies). We have tackled the indeterminacy of the problem by generating a family of solutions, although other strategies, based on data-re-sampling methods and supervised learning (similarly to what has been recently proposed by Huynh-Thu et al. [25]), might work well too. A systematic comparison of ensemble generation methods either based on problem structure or data re-sampling techniques should be considered in further work. Finally, a key point in the usage of ensemble methods is how to combine the models in order to obtain the best prediction possible. In this work we have chosen a simple model averaging framework. If more data become available, more sophisticated methods could be explored.

For the sake of computational reproducibility, we provide the SELDOM code as open source (http://doi.org/10.5281/zenodo.250558). This implementation can handle any data file in the MIDAS [51] format. Implementations for all the problems considered are also included in this distribution.

## Supporting information

S1 Text Ensemble time course trajectories for training data. (PDF)

S2 Text Ensemble time course trajectories for untested conditions(prediction data). (PDF)

S3 Text Relationship between the training and prediction RMSE for individual and ensemble models. For each case study we show scatter plots with the prediction RMSE as a function of the training RMSE for each individual model and the ensemble.
(PDF)

S4 TextEnsemble predictive skill depending on ensemble size for different case-studies.The curves shown in the Text were computed by bootstrapping multiple $n_{ℳ}$ models from the available 100 models, *i.e.* we sampled multiple realizations of the individual predictions for the same ensemble size and computed the average value. These curves converge asymptotically and show that the chosen ensemble size parameter is adequate. Equivalent predictions could have been obtained with smaller ensemble sizes.(PDF)

S5 TextAUPR score depending on ensemble size for different case-studies with and without applying model reduction.This curve was computed by bootstrapping multiple $n_{ℳ}$ models from the available 100 models, *i.e.* we sampled multiple realizations of the ensemble network for the same ensemble size and computed the average value.(PDF)

S6 Text AUPR curves for different algorithms and case-studies. (PDF)

S7 Text Networks obtained with SELDOM for different cell-lines of HPN-DREAM experimental data sub-challenge and discussion on the validity of the inferred interactions. (PDF)
