# Supplementary material for: Data-driven reverse engineering of signaling pathways using ensembles of dynamic models
Source: PLoS Comput Biol. 2017 Feb 6;13(2):e1005379. doi: 10.1371/journal.pcbi.1005379 (PMC5319798; doi:10.1371/journal.pcbi.1005379)
Supplement: S1 Text — (PDF) [file pcbi.1005379.s001.pdf]

# S1 Text: Ensemble time course trajectories obtained for training data

Data-driven reverse engineering of signaling pathways using ensembles of dynamic models

David Henriques, Alejandro F. Villaverde, Miguel Rocha, Julio Saez-Rodriguez, Julio R. Banga

January 17, 2017

# Contents

**1 Time-courses for training of different case-studies 3**
1.1 Case study 1a (MAPKp) . . . . . . . . . . . . . . . . . . . . . . . 3
1.2 Case study 1b (MAPKf) . . . . . . . . . . . . . . . . . . . . . . . 4
1.3 Case study 2 (SSP) . . . . . . . . . . . . . . . . . . . . . . . . . 5
1.4 Case study 3 (DREAMiS) . . . . . . . . . . . . . . . . . . . . . . 6
1.5 Case study 4a (DREAMBT20) . . . . . . . . . . . . . . . . . . . . 7
1.6 Case study 4b (DREAMBT549) . . . . . . . . . . . . . . . . . . . 9
1.7 Case study 4c (DREAMMCF7) . . . . . . . . . . . . . . . . . . . 11
1.8 Case study 4d (DREAMUACC812) . . . . . . . . . . . . . . . . . 11

# List of Figures

1 Ensemble time course trajectories for the case study 1a (MAPKp) (training data) . . . . . . . . . . . . . . . . . . . . . . . . . . . 3
2 Ensemble time course trajectories for the case study 1b (MAPKf) (training data) . . . . . . . . . . . . . . . . . . . . . . . . . . . 4
3 Ensemble time course trajectories for the case study 2 (SSP) (training data) . . . . . . . . . . . . . . . . . . . . . . . . . . . 5
4 Ensemble time course trajectories for the case study 3 (DREAMiS) (training data) . . . . . . . . . . . . . . . . . . . . . . . . . . . 6
5 Ensemble time course trajectories for the case study 4a (DREAMBT20) (training data, part 1) . . . . . . . . . . . . . . . . . . . . . . . 7
6 Ensemble time course trajectories for the case study 4a (DREAMBT20) (training data, part 2) . . . . . . . . . . . . . . . . . . . . . . . 8
7 Ensemble time course trajectories for the case study 4b (DREAMBT549) (training data, part 1) . . . . . . . . . . . . . . . . . . . . . . . 9
8 Ensemble time course trajectories for the case study 4b (DREAMBT549) (training data, part 2) . . . . . . . . . . . . . . . . . . . . . . . 10
9 Ensemble time course trajectories for the case study 4c (DREAMMCF7) (training data, part 1) . . . . . . . . . . . . . . . . . . . . . . . 11
10 Ensemble time course trajectories for the case study 4c (DREAMMCF7) (training data, part 2) . . . . . . . . . . . . . . . . . . . . . . . 12
11 Ensemble time course trajectories for the case study 4d (DREAMUACC812) (training data, part 1) . . . . . . . . . . . . . . . . 13
12 Ensemble time course trajectories for the case study 4d (DREAMUACC812) (training data, part 2) . . . . . . . . . . . . . . . . 14

# 1 Time-courses for training of different case-studies

## 1.1 Case study 1a (MAPKp)

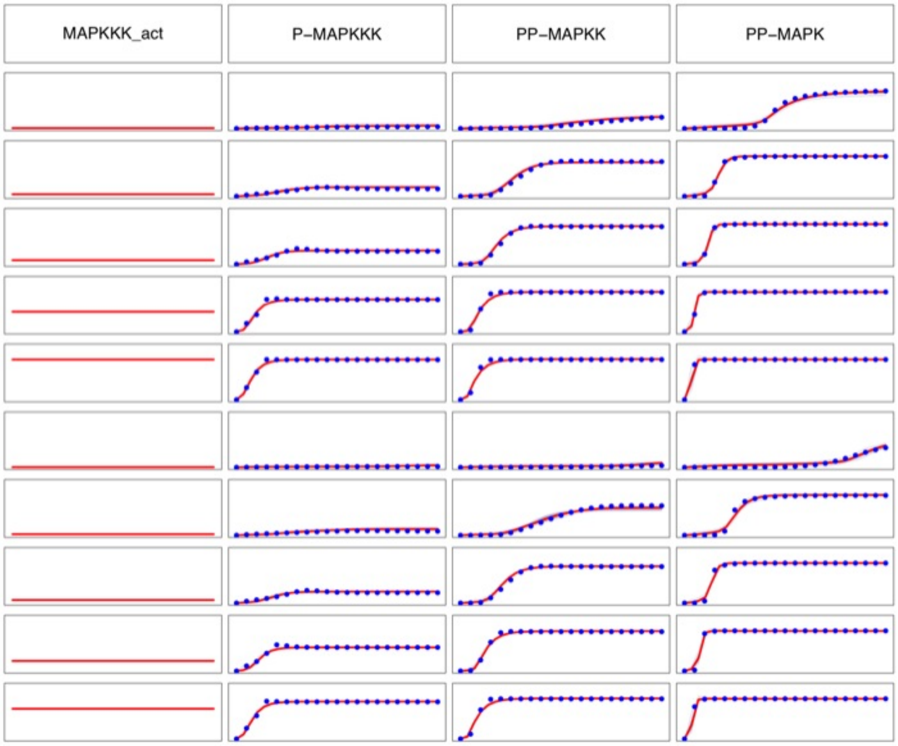


Figure 1: *Ensemble time course trajectories for the case study 1a (MAPKp) (training data)* The median in red is surrounded by the predicted non-symmetric 20% ,60% and 95%. Blue dots represent the pseudo-experimental data. Each row is an experiment and each column an observed signal.

## 1.2 Case study 1b (MAPKf)

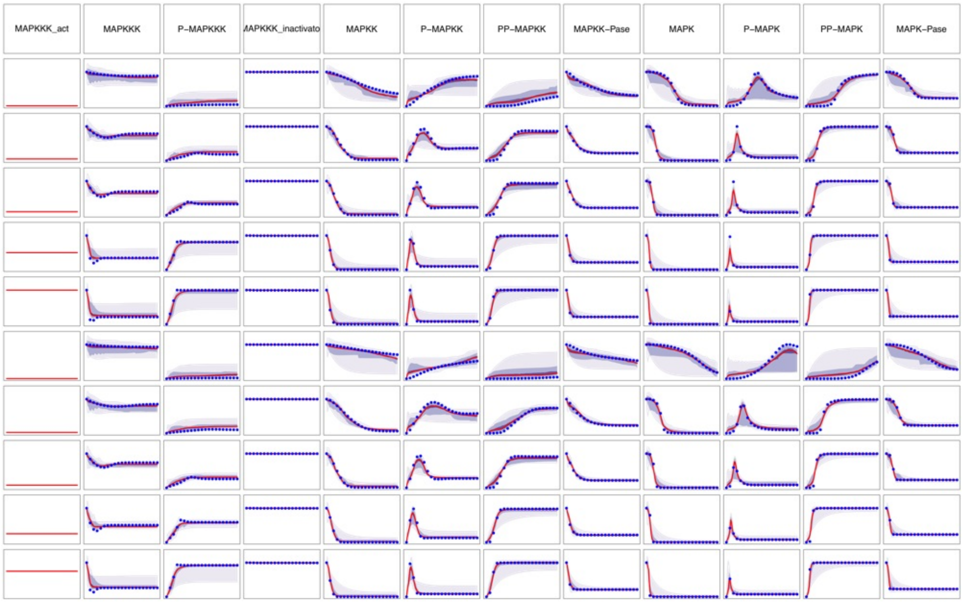


Figure 2: *Ensemble time course trajectories for the case study 1b (MAPKf) (training data)* The median in red is surrounded by the predicted non-symmetric 20% ,60% and 95%. Blue dots represent the pseudo-experimental data. Each row is an experiment and each column an observed signal.

## 1.3 Case study 2 (SSP)

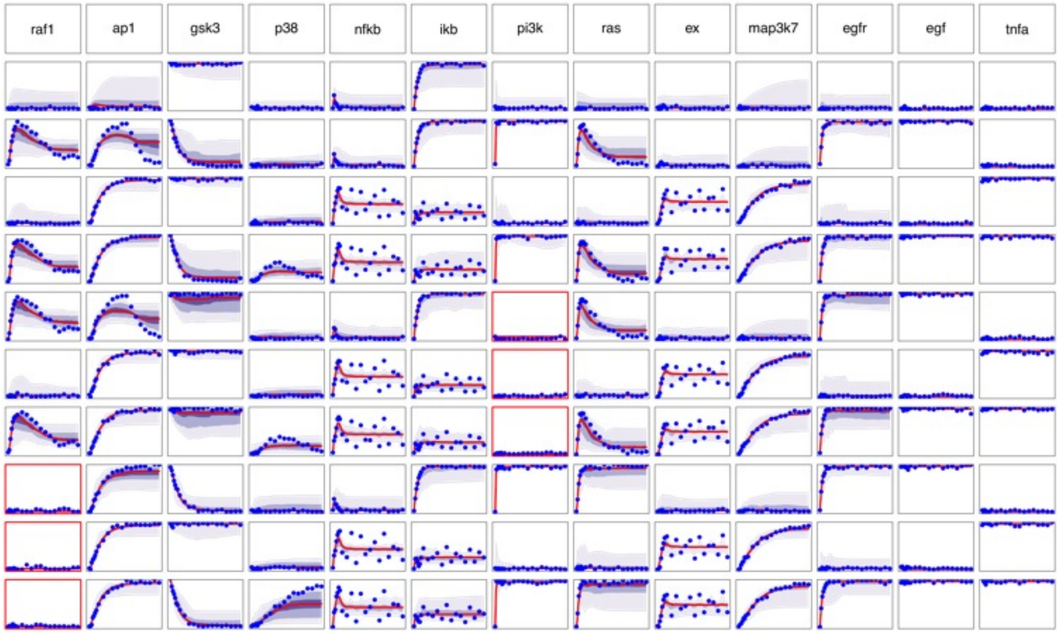


Figure 3: *Ensemble time course trajectories for the case study 2 (SSP) (training data)* The median in red is surrounded by the predicted non-symmetric 20% ,60% and 95%. Blue dots represent the pseudo-experimental data. Each row is an experiment and each column an observed signal.

## 1.4 Case study 3 (DREAMiS)

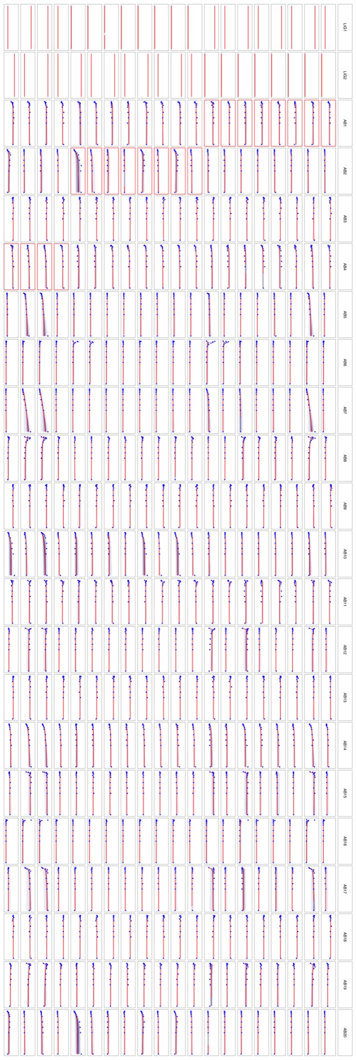

Figure 4: *Ensemble time course trajectories for the case study 2 (DREAMiS) (training data)* The median in red is surrounded by the predicted non-symmetric 20% ,60% and 95%. Blue dots represent the pseudo-experimental data. Each row is an experiment and each column an observed signal. Inhibited signals are surrounded by a red frame.

## 1.5 Case study 4a (DREAMBT20)

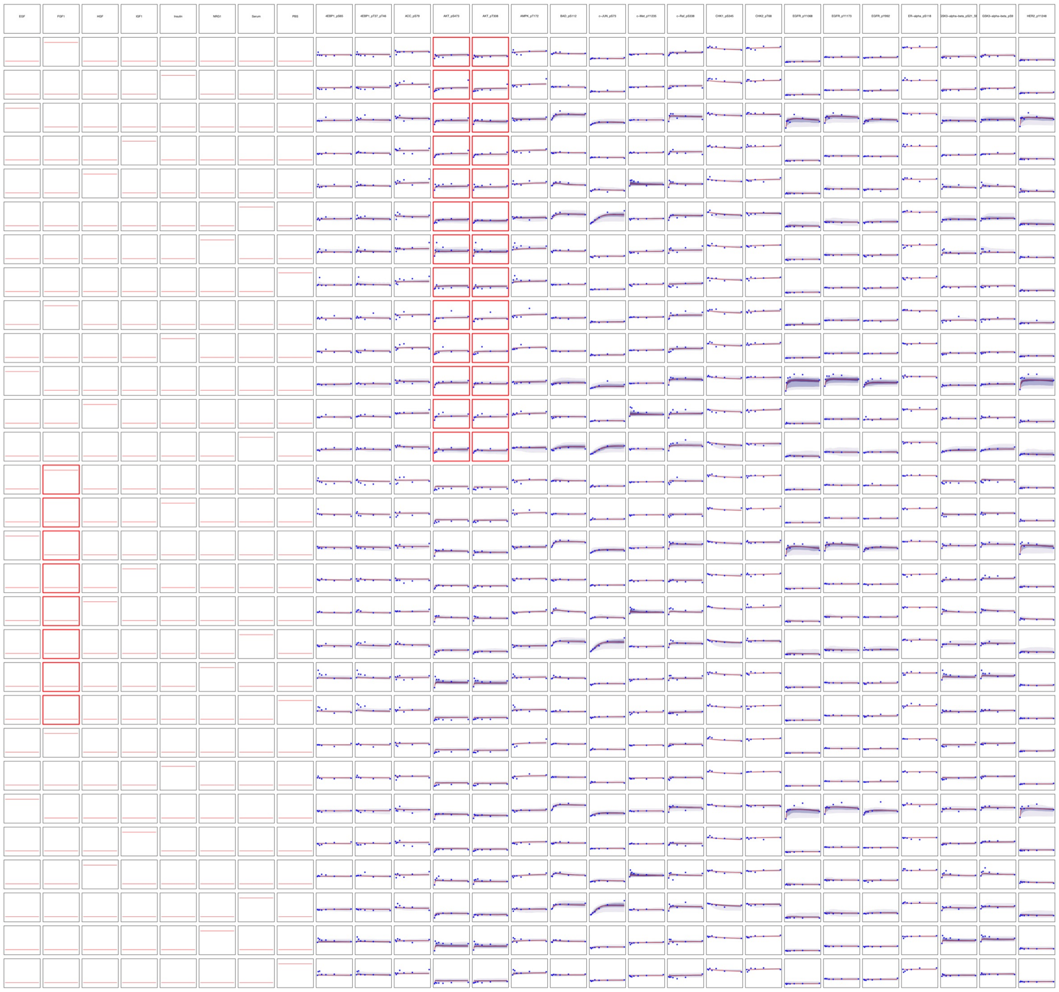

Figure 5: *Ensemble time course trajectories for the case study 4a (DREAMBT20) (training data, part 1)* The median in red is surrounded by the predicted non-symmetric 20% ,60% and 95%. Blue dots represent the experimental data. Each row is an experiment and each column an observed signal or stimuli. Inhibited signals are surrounded by a red frame and data for these experiments/signals combinations.

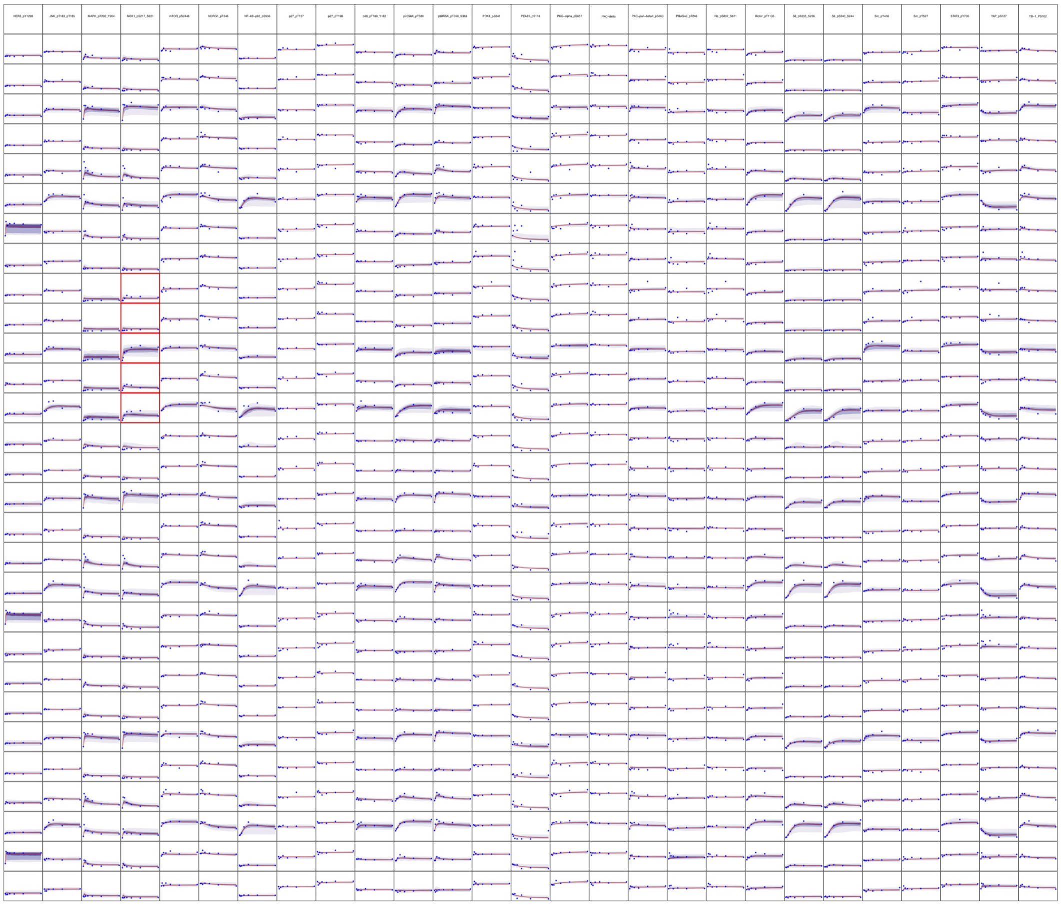

Figure 6: *Ensemble time course trajectories for the case study 4a (DREAMBT20) (training data, part 2)* The median in red is surrounded by the predicted non-symmetric 20% ,60% and 95%. Blue dots represent the experimental data. Each row is an experiment and each column an observed signal or stimuli. Inhibited signals are surrounded by a red frame and data for these experiments/signals combinations.

## 1.6 Case study 4b (DREAMBT549)

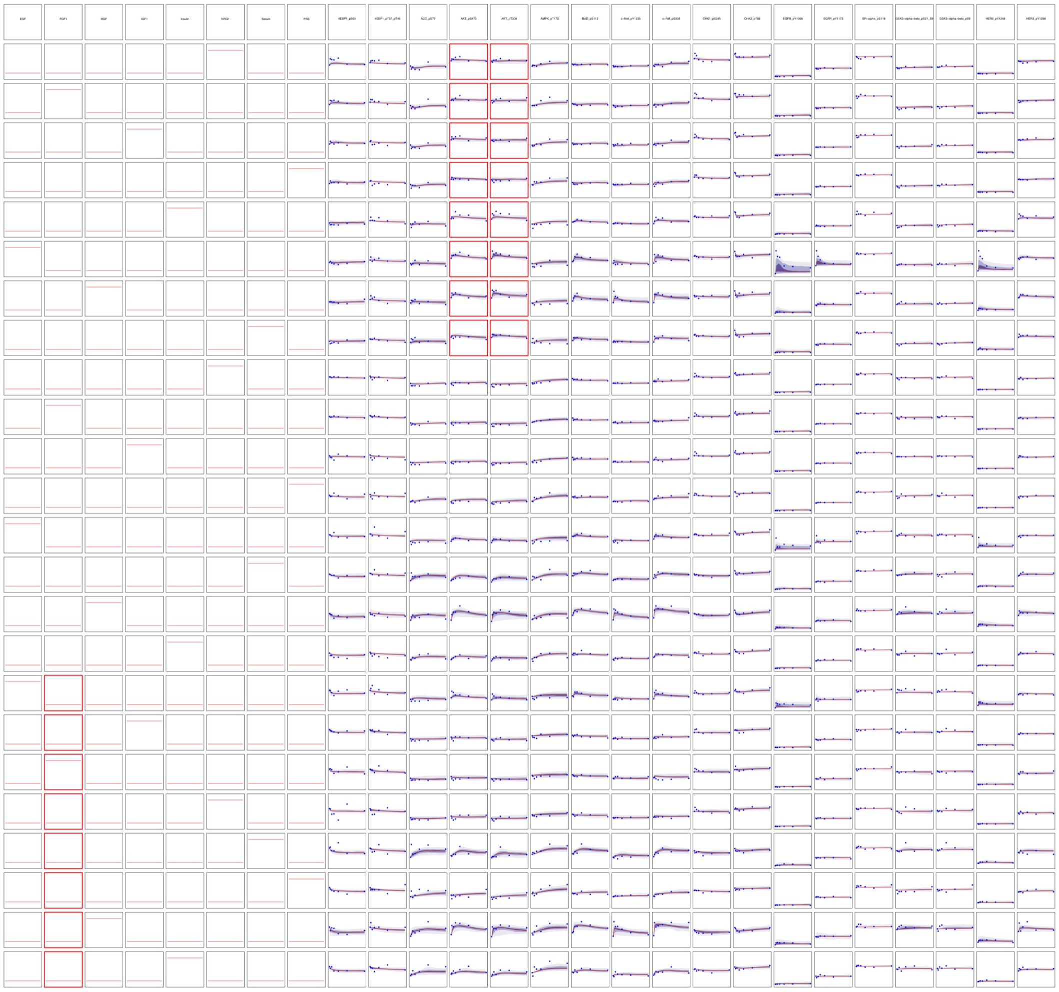

Figure 7: *Ensemble time course trajectories for the case study 4b (DREAMBT549) (training data, part 1)* The median in red is surrounded by the predicted non-symmetric 20% ,60% and 95%. Blue dots represent the experimental data. Each row is an experiment and each column an observed signal or stimuli. Inhibited signals are surrounded by a red frame and data for these experiments/signals combinations.

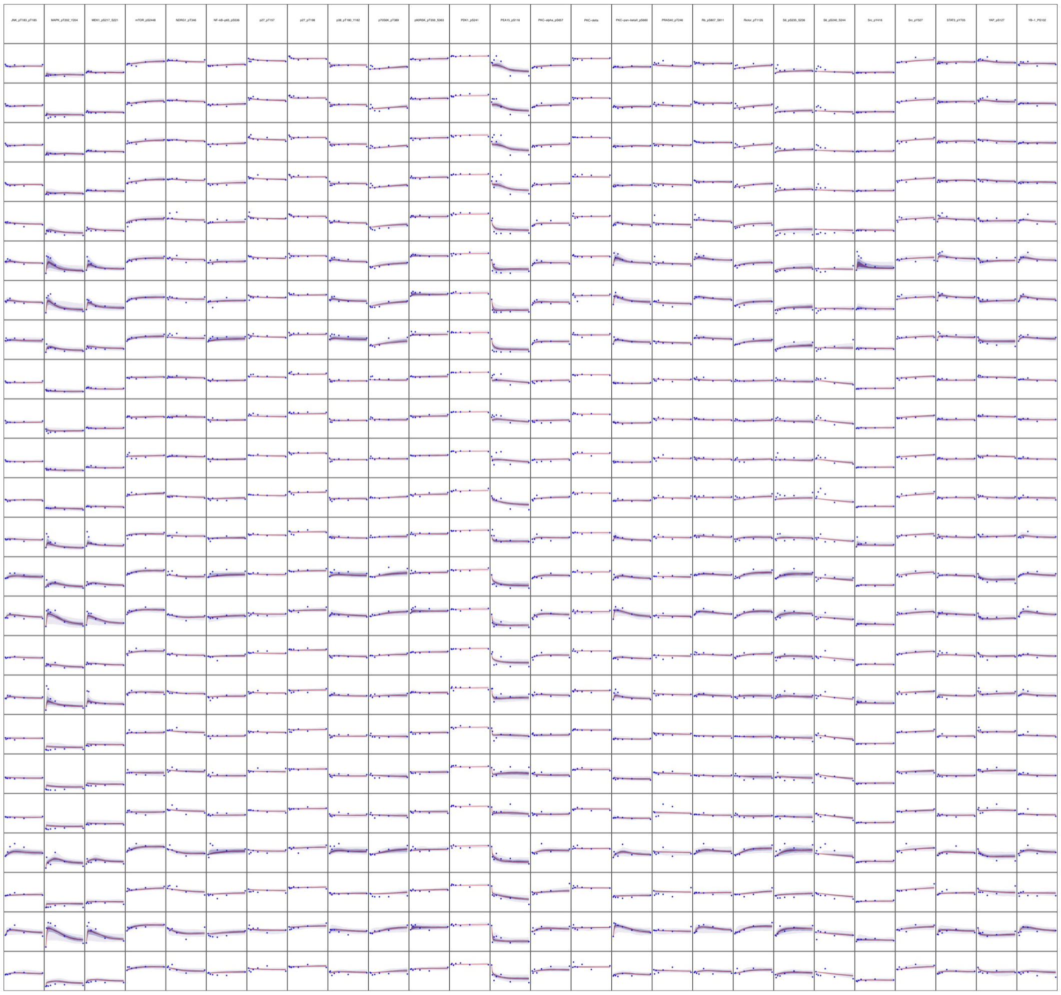

Figure 8: *Ensemble time course trajectories for the case study 4b (DREAMBT549) (training data, part 2)* The median in red is surrounded by the predicted non-symmetric 20% ,60% and 95%. Blue dots represent the experimental data. Each row is an experiment and each column an observed signal or stimuli. Inhibited signals are surrounded by a red frame and data for these experiments/signals combinations.

## 1.7 Case study 4c (DREAMMCF7)

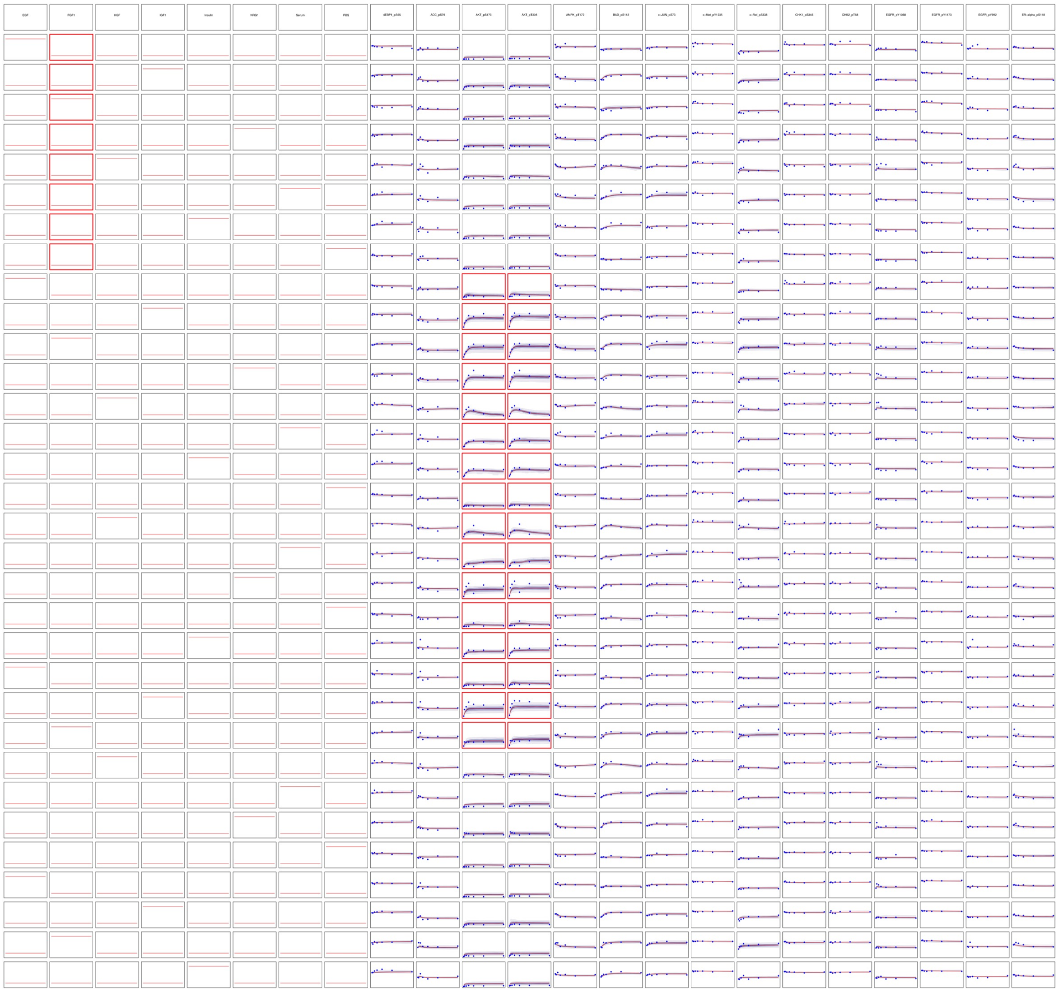

Figure 9: *Ensemble time course trajectories for the case study 4c (DREAMMCF7) (training data, part 1)* The median in red is surrounded by the predicted non-symmetric 20% ,60% and 95%. Blue dots represent the experimental data. Each row is an experiment and each column an observed signal or stimuli. Inhibited signals are surrounded by a red frame and data for these experiments/signals combinations.

## 1.8 Case study 4d (DREAMUACC812)

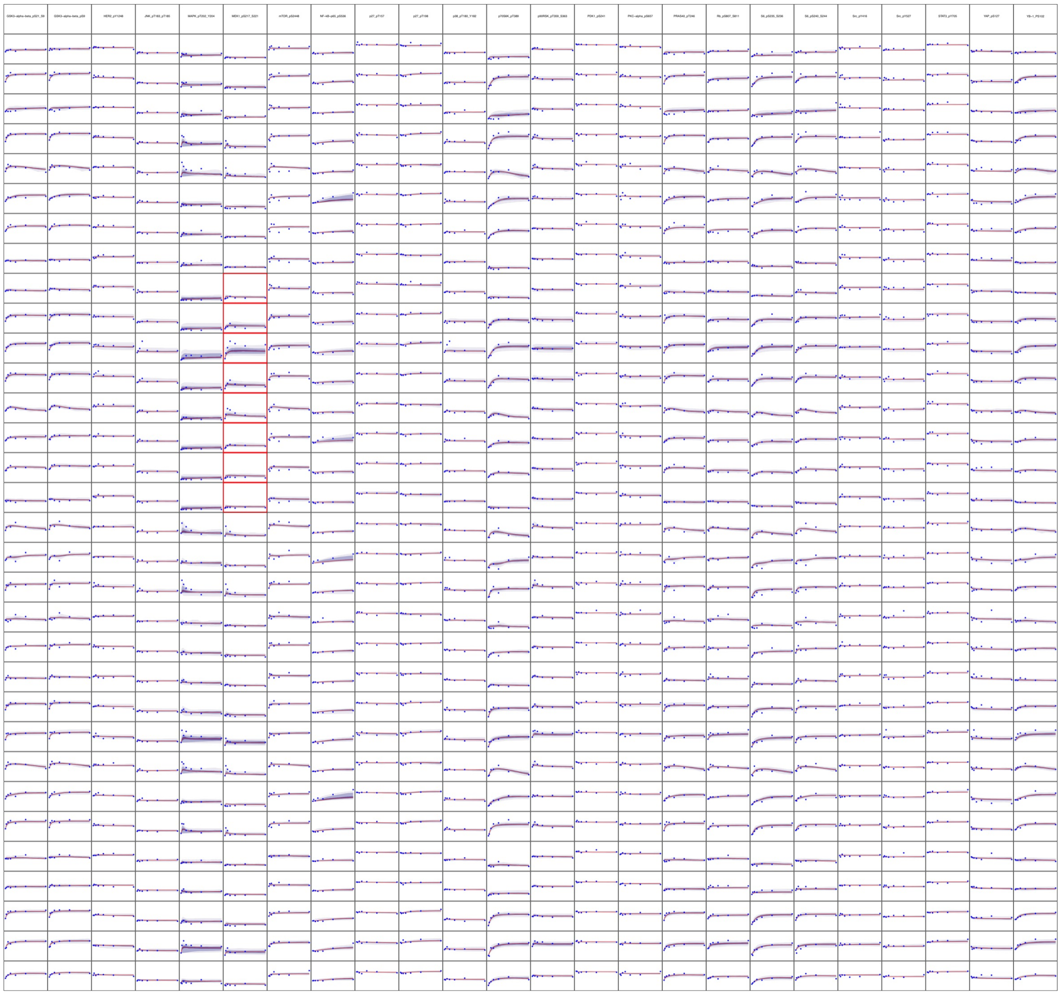

Figure 10: *Ensemble time course trajectories for the case study 4c (DREAMMCF7) (training data, part 2)* The median in red is surrounded by the predicted non-symmetric 20% ,60% and 95%. Blue dots represent the experimental data. Each row is an experiment and each column an observed signal or stimuli. Inhibited signals are surrounded by a red frame and data for these experiments/signals combinations.

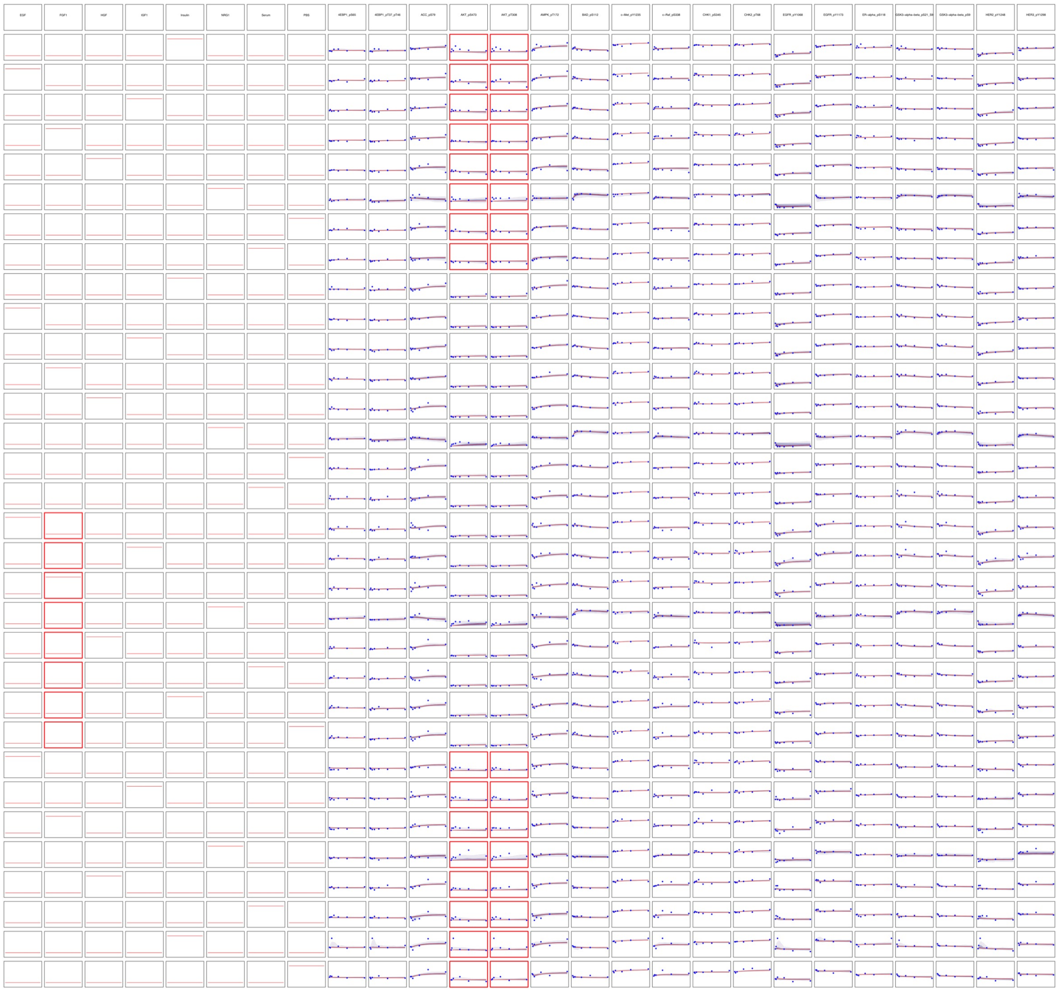

Figure 11: *Ensemble time course trajectories for the case study 4d (DREAM-UACC812) (training data, part 1)* The median in red is surrounded by the predicted non-symmetric 20% ,60% and 95%. Blue dots represent the experimental data. Each row is an experiment and each column an observed signal or stimuli. Inhibited signals are surrounded by a red frame and data for these experiments/signals combinations.

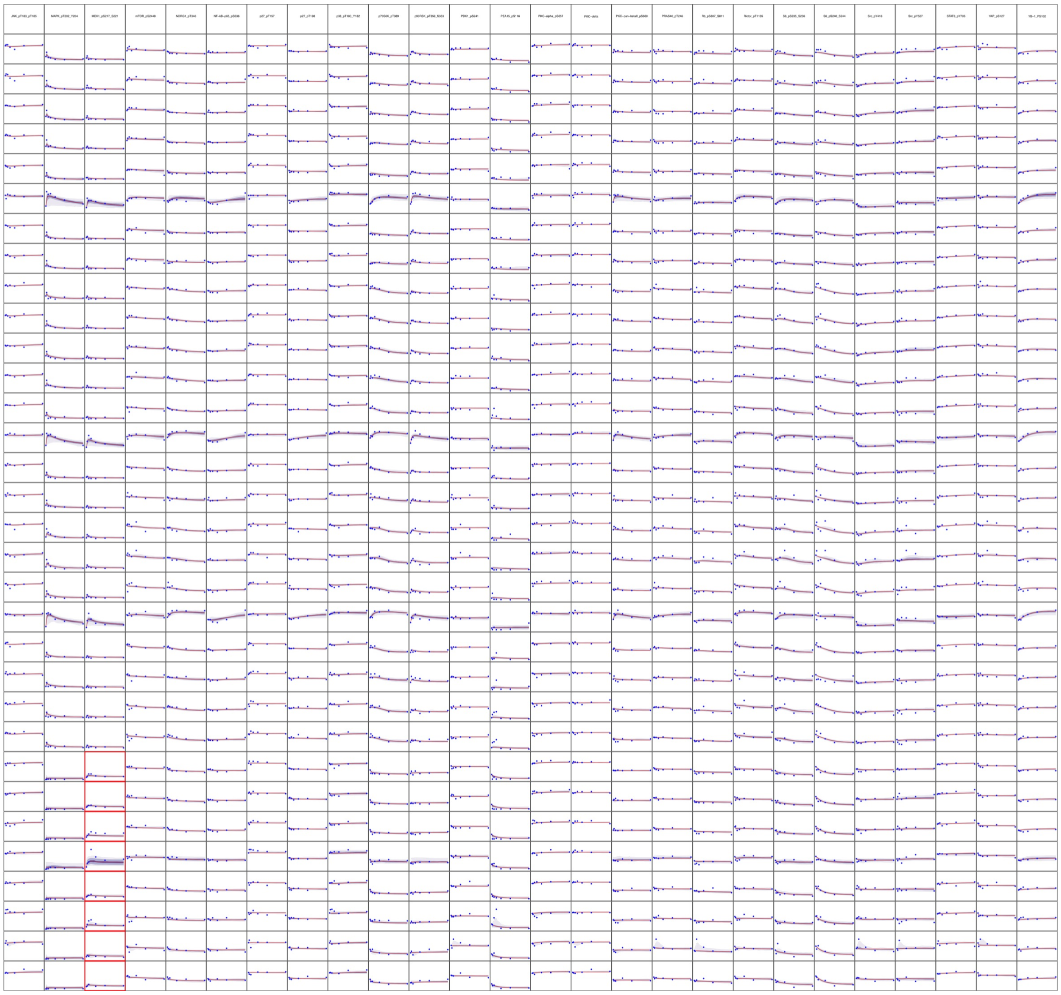

Figure 12: *Ensemble time course trajectories for the case study 4d (DREAM-UACC812) (training data, part 2)* The median in red is surrounded by the predicted non-symmetric 20% ,60% and 95%. Blue dots represent the experimental data. Each row is an experiment and each column an observed signal or stimuli. Inhibited signals are surrounded by a red frame and data for these experiments/signals combinations.
