# Supplementary material for: Data-driven reverse engineering of signaling pathways using ensembles of dynamic models
Source: PLoS Comput Biol. 2017 Feb 6;13(2):e1005379. doi: 10.1371/journal.pcbi.1005379 (PMC5319798; doi:10.1371/journal.pcbi.1005379)
Supplement: S2 Text — (PDF) [file pcbi.1005379.s002.pdf]

S2 Text: Ensemble time course trajectories obtained for prediction data

Data-driven reverse engineering of signaling pathways using ensembles of dynamic models

David Henriques, Alejandro F. Villaverde, Miguel Rocha, Julio Saez-Rodriguez, Julio R. Banga

January 17, 2017

Contents

1 Time-courses for prediction of different case-studies 3
1.1 Case study 1a (MAPKp) 3
1.2 Case study 1b (MAPKf) 4
1.3 Case study 2 (SSP) 5
1.4 Case study 3 (DREAMiS) 6
1.5 Case study 4a (DREAMBT20) 7
1.6 Case study 4b (DREAMBT549) 9
1.7 Case study 4c (DREAMMCF7) 11
1.8 Case study 4d (DREAMUACC812) 13

List of Figures

1 Ensemble time course trajectories for the case study 1a (MAPKp) (prediction data) 3
2 Ensemble time course trajectories for the case study 1b (MAPKf) (prediction data) 4
3 Ensemble time course trajectories for the case study 2 (SSP) (prediction data) 5
4 Ensemble time course trajectories for the case study 3 (DREAMiS) (prediction data) 6
5 Ensemble time course trajectories for the case study 4a (DREAMBT20) (training data, part 2) 7
6 Ensemble time course trajectories for the case study 4a (DREAMBT20) (prediction data, part 2) 8
7 Ensemble time course trajectories for the case study 4b (DREAMBT549) (prediction data, part 1) 9
8 Ensemble time course trajectories for the case study 4b (DREAMBT549) (prediction data, part 2) 10
9 Ensemble time course trajectories for the case study 4c (DREAMMCF7) (prediction data, part 1) 11
10 Ensemble time course trajectories for the case study 4c (DREAMMCF7) (prediction data, part 2) 12
11 Ensemble time course trajectories for the case study 4d (DREAMUACC812) (prediction data, part 1) 13
12 Ensemble time course trajectories for the case study 4d (DREAMUACC812) (prediction data, part 2) 14

1 Time-courses for prediction of different case-studies

1.1 Case study 1a (MAPKp)

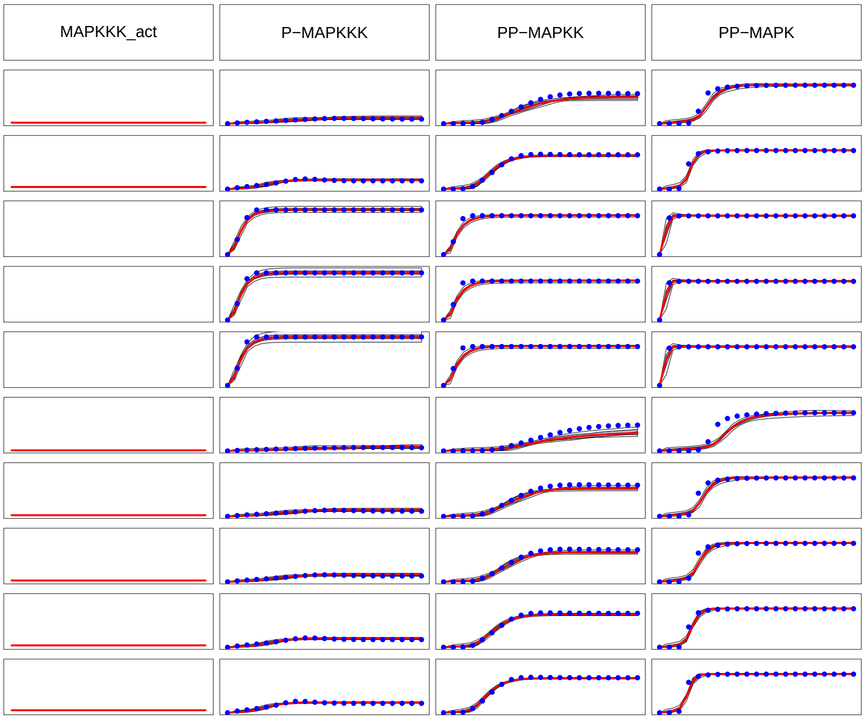

Figure 1: *Ensemble time course trajectories for the case study 1a (MAPKp) (prediction data)* The median in red is surrounded by the predicted non-symmetric 20% ,60% and 95%. Blue dots represent the pseudo-experimental data. Each row is a experiment and each column an observed signal.

1.2 Case study 1b (MAPKf)

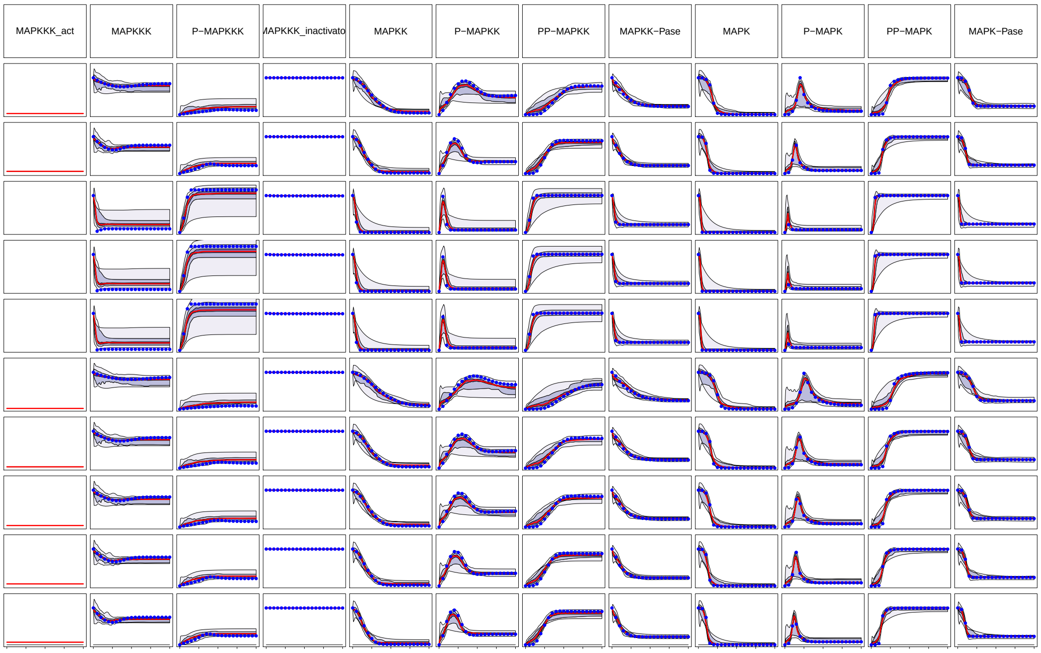

Figure 2: *Ensemble time course trajectories for the case study 1b (MAPKf) (prediction data)* The median in red is surrounded by the predicted non-symmetric 20% ,60% and 95%. Blue dots represent the pseudo-experimental data. Each row is a experiment and each column an observed signal.

1.3 Case study 2 (SSP)

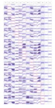

Figure 3: *Ensemble time course trajectories for the case study 2 (SSP) (prediction data)* The median in red is surrounded by the predicted non-symmetric 20% ,60% and 95%. Blue dots represent the pseudo-experimental data. Each row is a experiment and each column an observed signal. Inhibited signals are surrounded by a red frame and data for these experiments/signals combinations is omitted.

1.4 Case study 3 (DREAMiS)

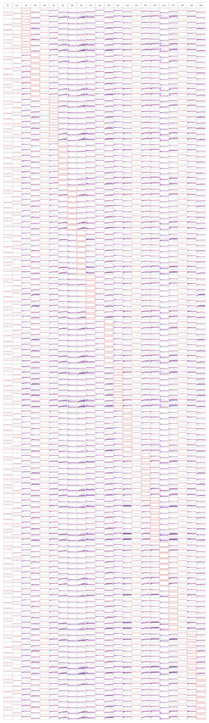

Figure 4: *Ensemble time course trajectories for the case study 3 (DREAMiS) (prediction data)* The median in red is surrounded by the predicted non-symmetric 20% ,60% and 95%. Blue dots represent the pseudo-experimental data. Each row is a experiment and each column an observed signal. Inhibited signals are surrounded by a red frame and data for these experiments/signals combinations is omitted. The magenta line shows the predictions made by Team34, the top performing team in the time-course prediction with *in silico* data DREAM-HPN sub-challenge.

1.5 Case study 4a (DREAMBT20)

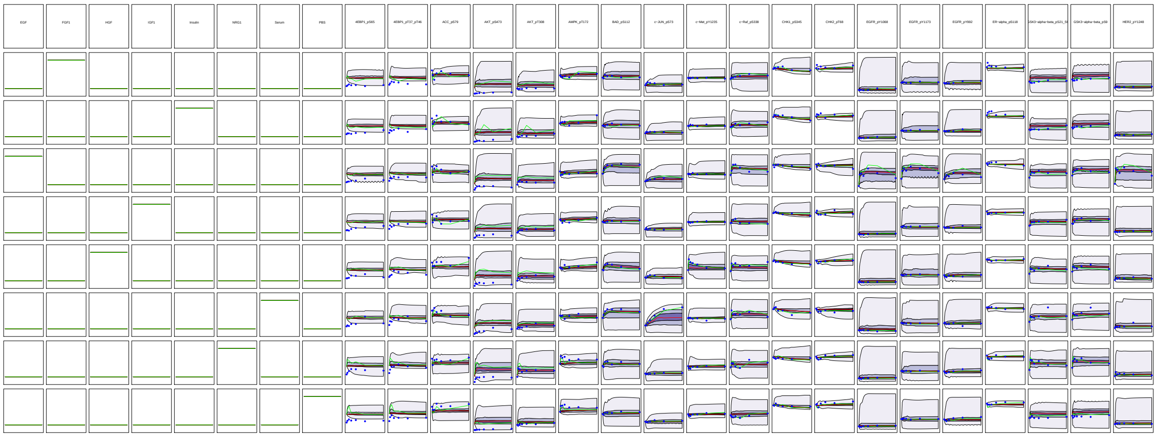

Figure 5: *Ensemble time course trajectories for the case study 4a (DREAMBT20) (training data, part 2)* The median in red is surrounded by the predicted non-symmetric 20% ,60% and 95%. Blue dots represent the experimental data. Each row is a experiment and each column an observed signal or stimuli. Inhibited signals are surrounded by a red frame and data for these experiments/signals combinations is shown but was not taken into account while computing root mean square error scores.

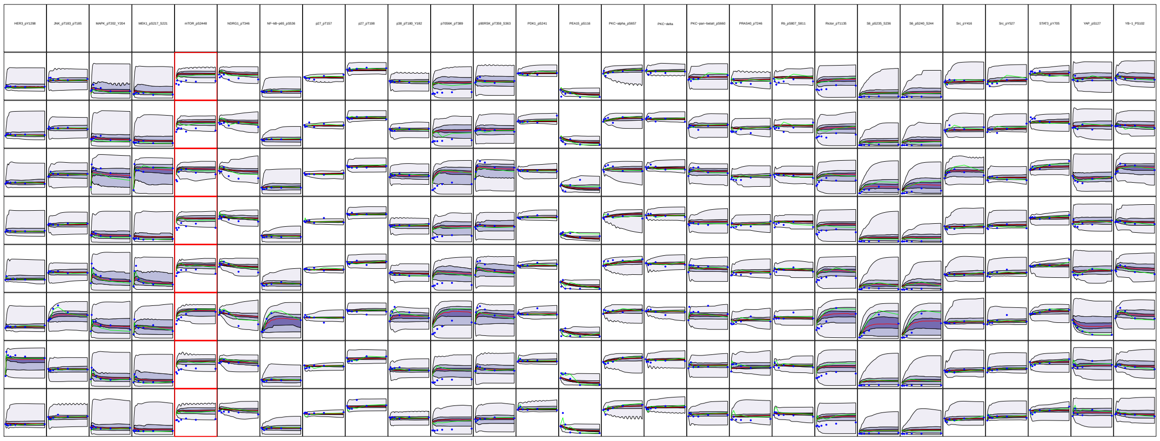

Figure 6: *Ensemble time course trajectories for the case study 4a (DREAMBT20) (prediction data, part 2)* The median in red is surrounded by the predicted non-symmetric 20% ,60% and 95%. Blue dots represent the experimental data. Each row is a experiment and each column an observed signal or stimuli. Inhibited signals are surrounded by a red frame and data for these experiments/signals combinations is shown but was not taken into account while computing root mean square error scores. The green line shows the predictions made by Team44, the top performing team in the time-course prediction with experimental data DREAM-HPN sub-challenge.

1.6 Case study 4b (DREAMBT549)

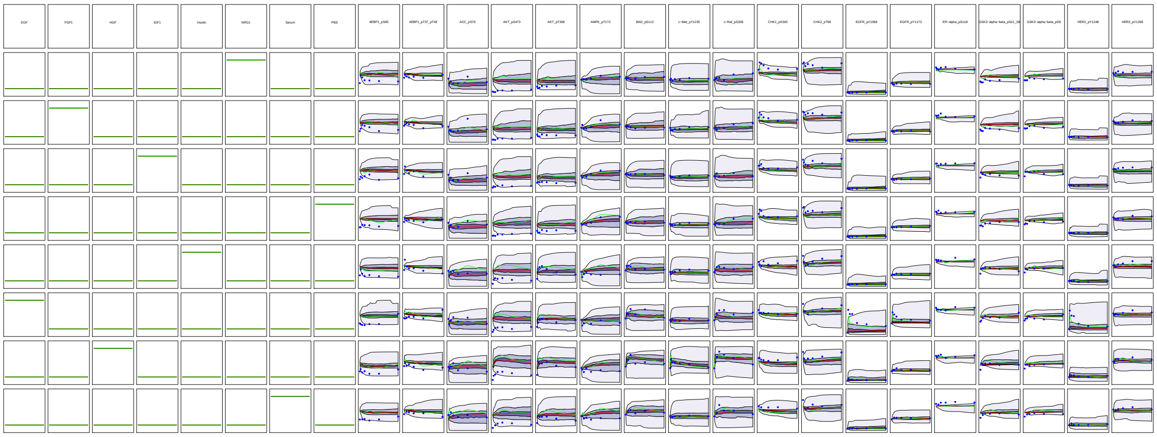

Figure 7: *Ensemble time course trajectories for the case study 4b (DREAMBT549) (prediction data, part 1)* The median in red is surrounded by the predicted non-symmetric 20% ,60% and 95%. Blue dots represent the experimental data. Each row is a experiment and each column an observed signal or stimuli. Inhibited signals are surrounded by a red frame and data for these experiments/signals combinations is shown but was not taken into account while computing root mean square error scores. The green line shows the predictions made by Team44, the top performing team in the time-course prediction with experimental data DREAM-HPN sub-challenge.

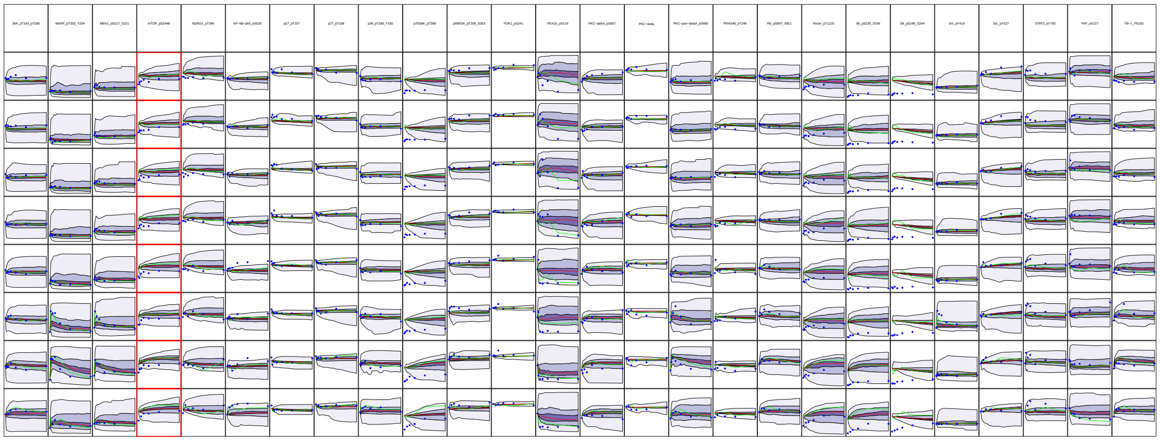

Figure 8: *Ensemble time course trajectories for the case study 4b (DREAMBT549) (prediction data, part 2)* The median in red is surrounded by the predicted non-symmetric 20% ,60% and 95%. Blue dots represent the experimental data. Each row is a experiment and each column an observed signal or stimuli. Inhibited signals are surrounded by a red frame and data for these experiments/signals combinations is shown but was not taken into account while computing root mean square error scores. The green line shows the predictions made by Team44, the top performing team in the time-course prediction with experimental data DREAM-HPN sub-challenge.

1.7 Case study 4c (DREAMMCF7)

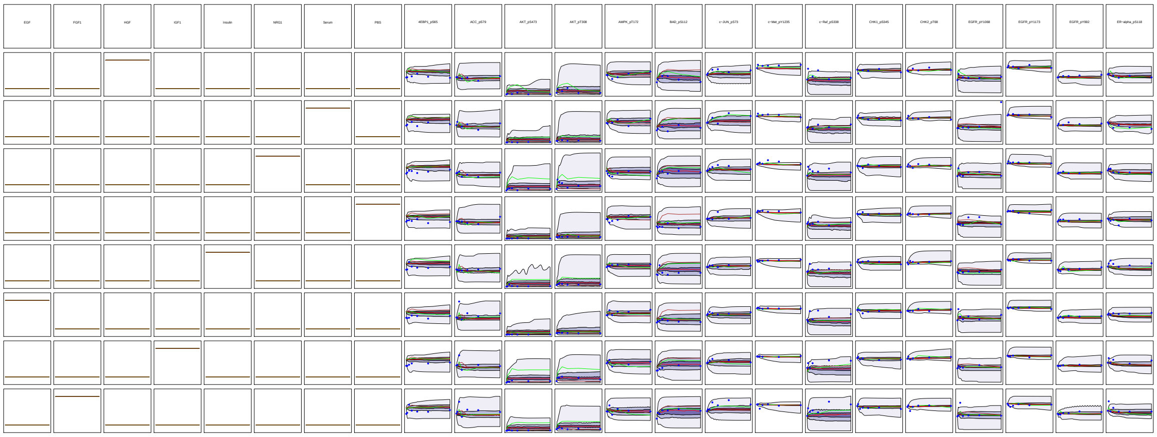

Figure 9: *Ensemble time course trajectories for the case study 4c (DREAMMCF7) (prediction data, part 1)* The median in red is surrounded by the predicted non-symmetric 20% ,60% and 95%. Blue dots represent the experimental data. Each row is a experiment and each column an observed signal or stimuli. Inhibited signals are surrounded by a red frame and data for these experiments/signals combinations is shown but was not taken into account while computing root mean square error scores. The green line shows the predictions made by Team44, the top performing team in the time-course prediction with experimental data DREAM-HPN sub-challenge.

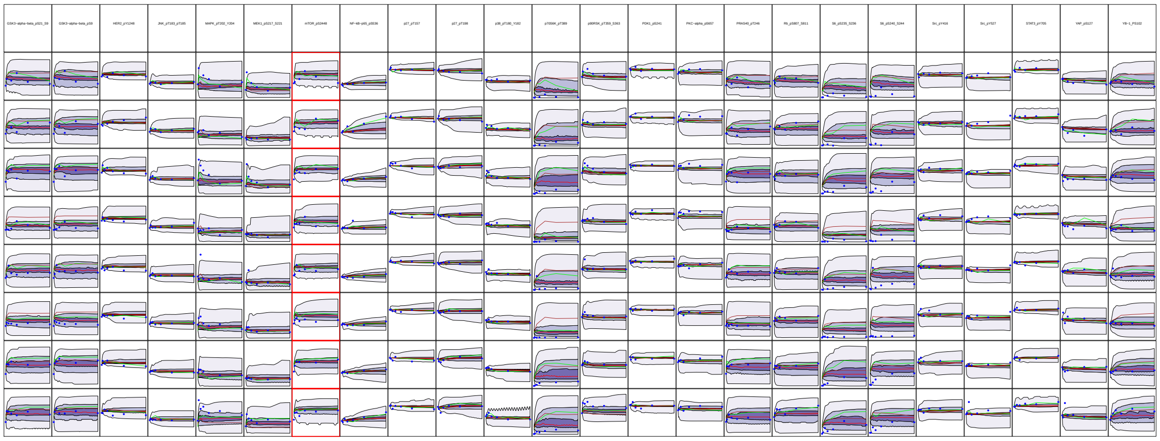

Figure 10: *Ensemble time course trajectories for the case study 4c (DREAMMCF7) (prediction data, part 2)* The median in red is surrounded by the predicted non-symmetric 20% ,60% and 95%. Blue dots represent the experimental data. Each row is a experiment and each column an observed signal or stimuli. Inhibited signals are surrounded by a red frame and data for these experiments/signals combinations is shown but was not taken into account while computing root mean square error scores. The green line shows the predictions made by Team44, the top performing team in the time-course prediction with experimental data DREAM-HPN sub-challenge.

1.8 Case study 4d (DREAMUACC812)

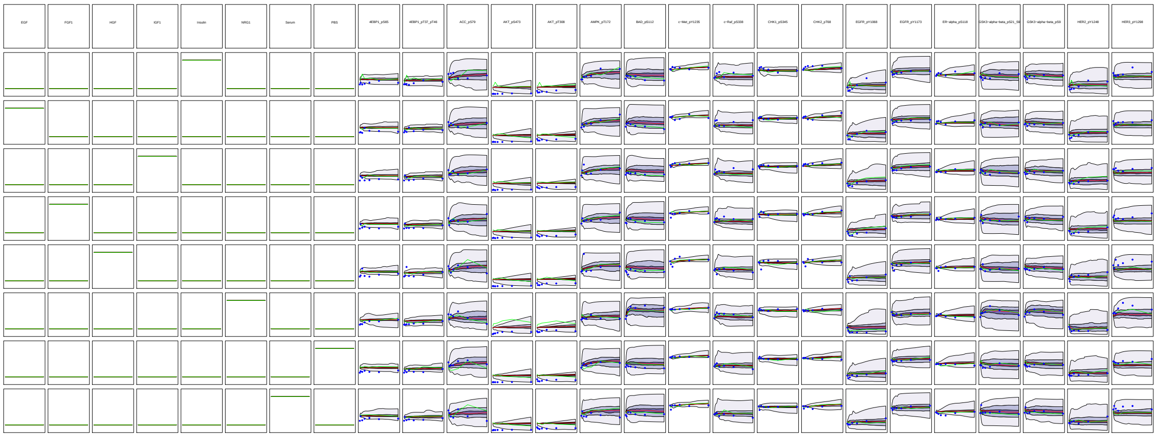

Figure 11: *Ensemble time course trajectories for the case study 4d (DDREAMUACC812) (prediction data, part 1)* The median in red is surrounded by the predicted non-symmetric 20% ,60% and 95%. Blue dots represent the experimental data. Each row is a experiment and each column an observed signal or stimuli. Inhibited signals are surrounded by a red frame and data for these experiments/signals combinations is shown but was not taken into account while computing root mean square error scores. The green line shows the predictions made by Team44, the top performing team in the time-course prediction with experimental data DREAM-HPN sub-challenge.

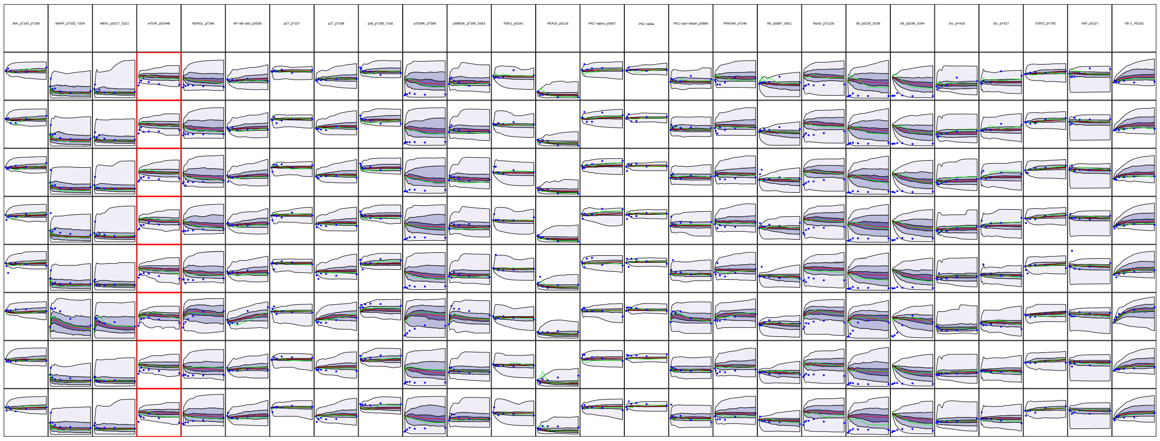

Figure 12: *Ensemble time course trajectories for the case study 4d (DDREA-MUACC812) (prediction data, part 2)* The median in red is surrounded by the predicted non-symmetric 20% ,60% and 95%. Blue dots represent the experimental data. Each row is a experiment and each column an observed signal or stimuli. Inhibited signals are surrounded by a red frame and data for these experiments/signals combinations is shown but was not taken into account while computing root mean square error scores. The green line shows the predictions made by Team44, the top performing team in the time-course prediction with experimental data DREAM-HPN sub-challenge.
