# Supplementary material for: Data-driven reverse engineering of signaling pathways using ensembles of dynamic models
Source: PLoS Comput Biol. 2017 Feb 6;13(2):e1005379. doi: 10.1371/journal.pcbi.1005379 (PMC5319798; doi:10.1371/journal.pcbi.1005379)
Supplement: S3 Text — For each case study we show scatter plots with the prediction RMSE as a function of the training RMSE for each individual model and the ensemble. (PDF) [file pcbi.1005379.s003.pdf]

S3 Text: Relationship between training and prediction RMSE for individual and ensemble models

Data-driven reverse engineering of signaling pathways using ensembles of dynamic models

David Henriques, Alejandro F. Villaverde, Miguel Rocha, Julio Saez-Rodriguez, Julio R. Banga

January 17, 2017

Contents

1 **Relationship between the training and prediction RMSE for individual and ensemble models** **3**
1.1 Case study 1a (MAPKp) 3
1.2 Case study 1b (MAPKf) 4
1.3 Case study 2 (SSP) 5
1.4 Case study 3 (DREAMiS) 6
1.5 Case study 4a (DREAMBT20) 7
1.6 Case study 4b (DREAMBT549) 8
1.7 Case study 4c (DREAMMCF7) 9
1.8 Case study 4d (DREAMUACC812) 10

List of Figures

1 Relationship between training and prediction RMSE for case study 1a (MAPFp) 3
2 Relationship between training and prediction RMSE for case study 1b (MAPFf) 4
3 Relationship between training and prediction RMSE for case study 2 (SSP) 5
4 Relationship between training and prediction RMSE for case study 3 (DREAMiS) 6
5 Relationship between training and prediction RMSE for case study 4a (DREAMBT20) 7
6 Relationship between training and prediction RMSE for case study 4b (DREAMBT549) 8
7 Relationship between training and prediction RMSE for case study 4c (DREAMMCF7) 9
8 Relationship between training and prediction RMSE for case study 4d (DREAMUACC812) 10

1 Relationship between the training and prediction RMSE for individual and ensemble models

1.1 Case study 1a (MAPKp)

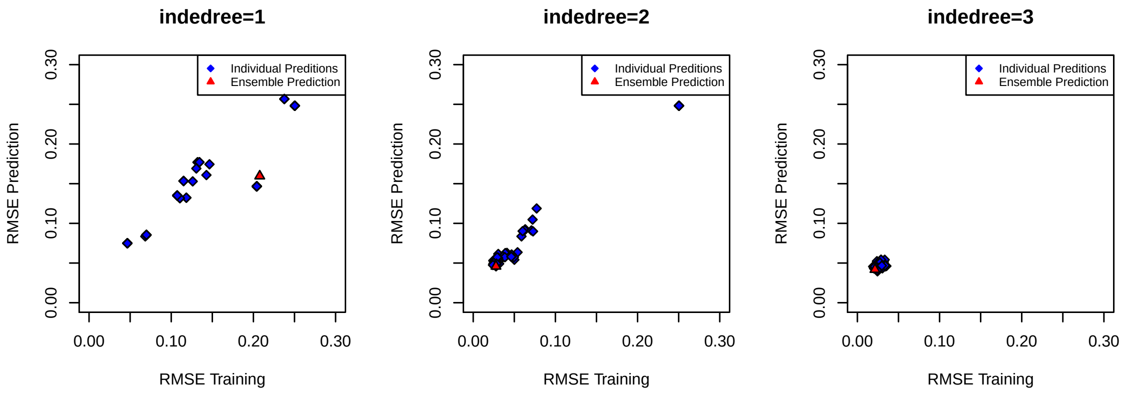

Figure 1: *Relationship between training and prediction RMSE for case study 1a (MAPFp).* The prediction RMSE is plotted here against the training RMSE for each individual model (blue) and the ensemble (red).

1.2 Case study 1b (MAPKf)

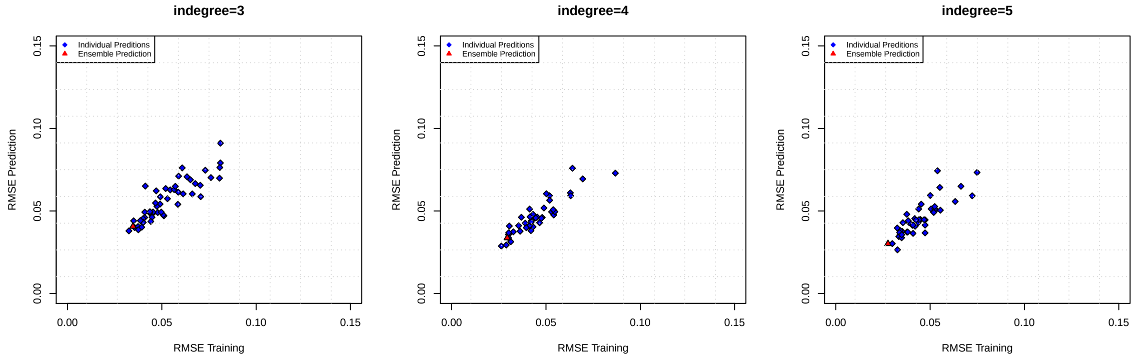

Figure 2: *Relationship between training and prediction RMSE for case study 1b (MAPFf).* The prediction RMSE is plotted here against the training RMSE for each individual model (blue) and the ensemble (red).

1.3 Case study 2 (SSP)

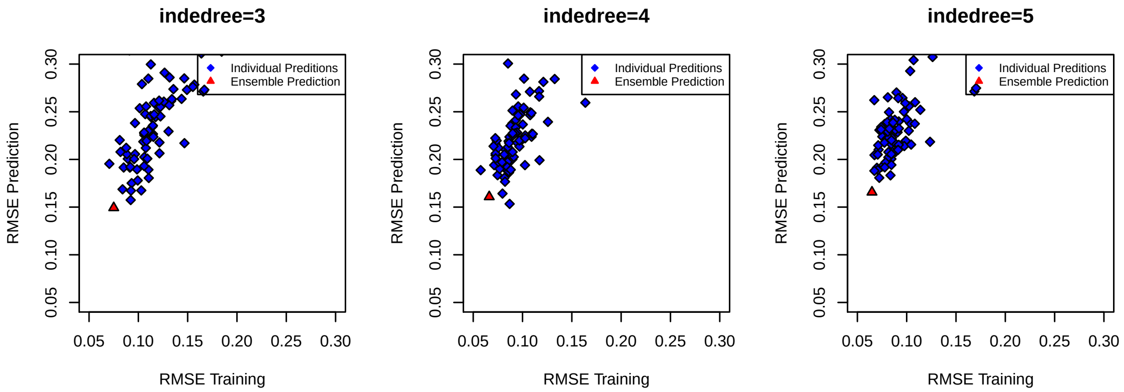

Figure 3: *Relationship between training and prediction RMSE for case study 2 (SSP).* The prediction RMSE is plotted here against the training RMSE for each individual model (blue) and the ensemble (red).

1.4 Case study 3 (DREAMiS)

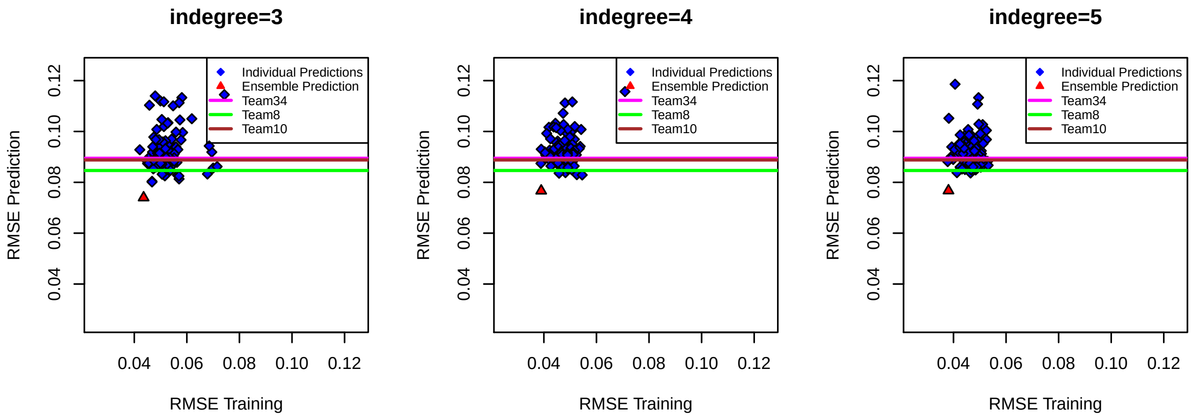

Figure 4: *Relationship between training and prediction RMSE for case study 3 (DREAMiS).* The prediction RMSE is plotted here against the training RMSE for each individual model (blue) and the ensemble (red). RMSE scores for the top 3 performing teams in the HPN-DREAM *in silico* time-course prediction sub-challenge (Team34, Team8 and Team10) are also shown colored lines.

1.5 Case study 4a (DREAMBT20)

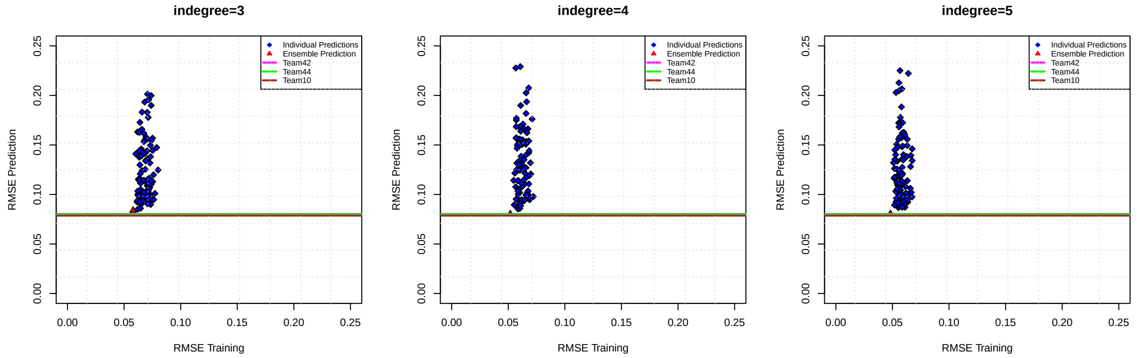

Figure 5: *Relationship between training and prediction RMSE for case study 4a (DREAMBT20).* The prediction RMSE is plotted here against the training RMSE for each individual model (blue) and the ensemble (red). RMSE scores for the top 3 performing teams in the HPN-DREAM experimental time-course prediction sub-challenge (Team44, Team42 and Team10) are also shown colored lines.

1.6 Case study 4b (DREAMBT549)

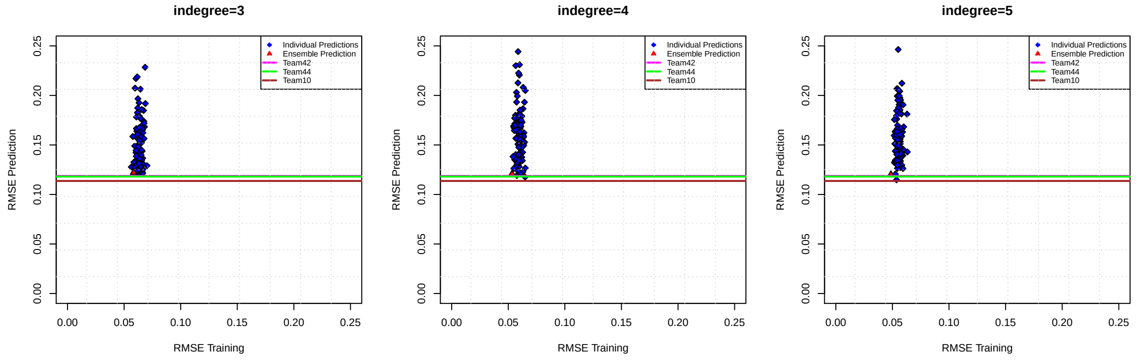

Figure 6: *Relationship between training and prediction RMSE for case study 4b (DREAMBT549).* The prediction RMSE is plotted here against the training RMSE for each individual model (blue) and the ensemble (red). RMSE scores for the top 3 performing teams in the HPN-DREAM experimental time-course prediction sub-challenge (Team44, Team42 and Team10) are also shown colored lines.

1.7 Case study 4c (DREAMMCF7)

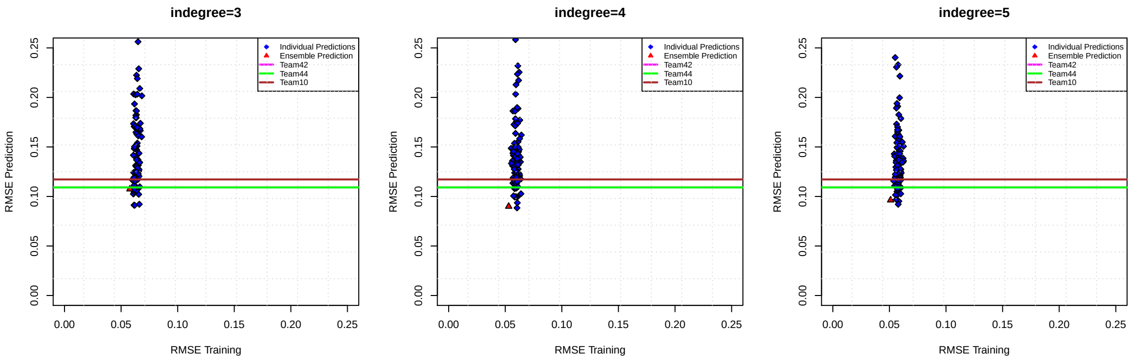

Figure 7: *Relationship between training and prediction RMSE for case study 4c (DREAMMCF7).* The prediction RMSE is plotted here against the training RMSE for each individual model (blue) and the ensemble (red). RMSE scores for the top 3 performing teams in the HPN-DREAM experimental time-course prediction sub-challenge (Team44, Team42 and Team10) are also shown colored lines.

1.8 Case study 4d (DREAMUACC812)

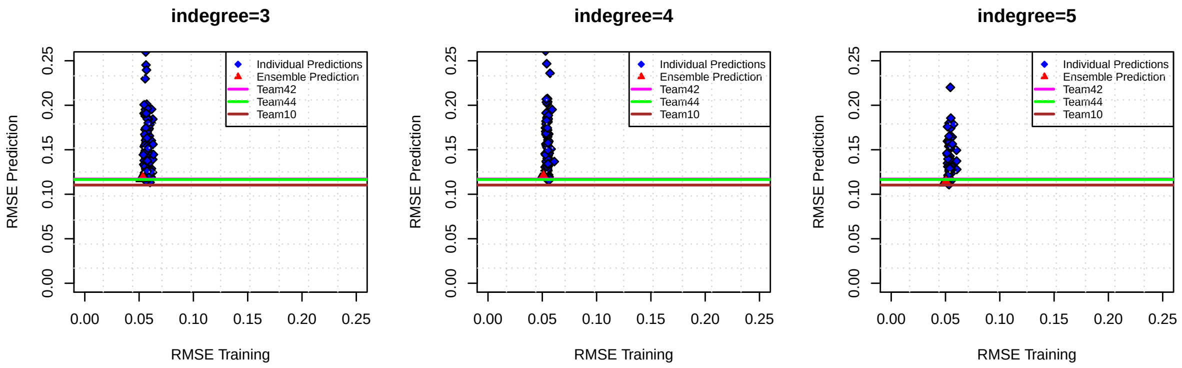

Figure 8: *Relationship between training and prediction RMSE for case study 4d (DREAMUACC812).* The prediction RMSE is plotted here against the training RMSE for each individual model (blue) and the ensemble (red). RMSE scores for the top 3 performing teams in the HPN-DREAM experimental time-course prediction sub-challenge (Team44, Team42 and Team10) are also shown colored lines.
