# Supplementary material for: Data-driven reverse engineering of signaling pathways using ensembles of dynamic models
Source: PLoS Comput Biol. 2017 Feb 6;13(2):e1005379. doi: 10.1371/journal.pcbi.1005379 (PMC5319798; doi:10.1371/journal.pcbi.1005379)
Supplement: S4 Text — The curves shown in the Text were computed by bootstrapping multiple nℳ models from the available 100 models, i.e. we sampled multiple realizations of the individual predictions for the same ensemble size and computed the average value. These curves converge asymptotically and show that the chosen ensemble size parameter is adequate. Equivalent predictions could have been obtained with smaller ensemble sizes. (PDF) [file pcbi.1005379.s004.pdf]

S4 Text: Ensemble predictive skill depending on ensemble size for different case-studies

Data-driven reverse engineering of signaling pathways using ensembles of dynamic models

David Henriques, Alejandro F. Villaverde, Miguel Rocha,
Julio Saez-Rodriguez, Julio R. Banga

January 17, 2017

Contents

1 Ensemble predictive skill depending on ensemble size for different case-studies 3
- 1.1 Case study 1a (MAPKp) 3
- 1.2 Case study 1b (MAPKf) 4
- 1.3 Case study 2 (SSP) 5
- 1.4 Case study 3 (DREAMiS) 6
- 1.5 Case study 4a (DREAMBT20) 7
- 1.6 Case study 4b (DREAMBT549) 8
- 1.7 Case study 4c (DREAMMCF7) 9
- 1.8 Case study 4d (DREAMUACC812) 10

List of Figures

1 Ensemble predictive skill depending on ensemble size for case study 1a (MAPKp) 3
2 Ensemble predictive skill depending on ensemble size for case study 1b (MAPKf) 4
3 Ensemble predictive skill depending on ensemble size for case study 2 (SSP) 5
4 Ensemble predictive skill depending on ensemble size for case study 3 (DREAMiS) 6
5 Ensemble predictive skill depending on ensemble size for case study 4a (DREAMBT20) 7
6 Ensemble predictive skill depending on ensemble size for case study 4b (DREAMBT549) 8
7 Ensemble predictive skill depending on ensemble size for case study 4c (DREAMMCF7) 9
8 Ensemble predictive skill depending on ensemble size for case study 4d (DREAMUACC812) 10

1 Ensemble predictive skill depending on ensemble size for different case-studies

1.1 Case study 1a (MAPKp)

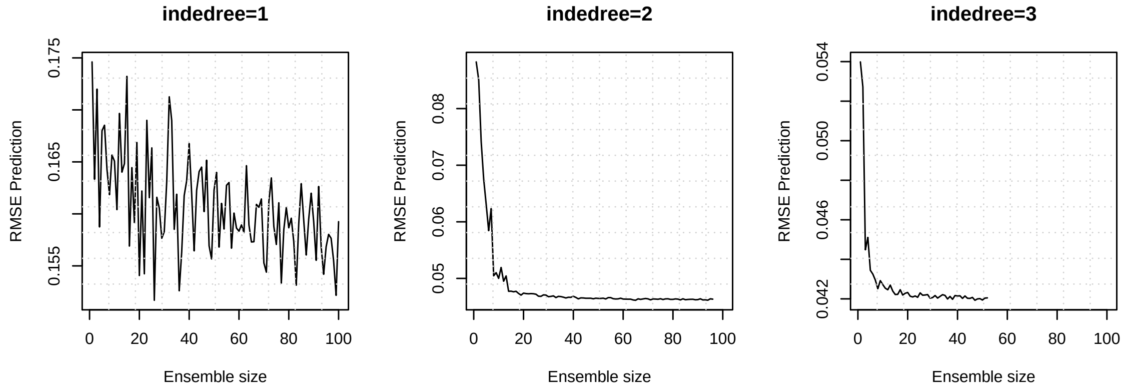

Figure 1: *Ensemble predictive skill depending on ensemble size for case study 1a (MAPKp).* The curve was computed by bootstrapping multiple $n_{\mathcal{M}}$ models from the available models, *i.e.* we sampled multiple realizations of the individual predictions for the same ensemble size and computed the average value. These curves converge asymptotically and show that the chosen ensemble size parameter is adequate. Equivalent predictions could have been obtained with smaller ensemble sizes.

1.2 Case study 1b (MAPKf)

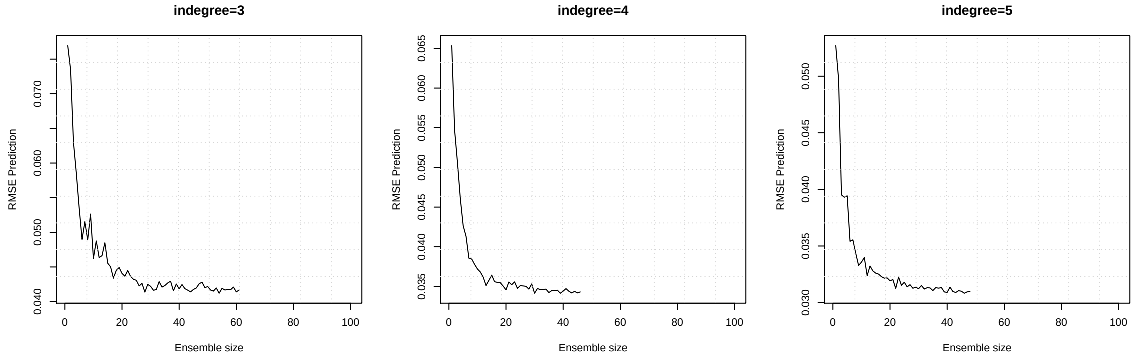

Figure 2: *Ensemble predictive skill depending on ensemble size for case study 1b (MAPKf).* The curve was computed by bootstrapping multiple $n_{\mathcal{M}}$ models from the available models, *i.e.* we sampled multiple realizations of the individual predictions for the same ensemble size and computed the average value. These curves converge asymptotically and show that the chosen ensemble size parameter is adequate. Equivalent predictions could have been obtained with smaller ensemble sizes.

1.3 Case study 2 (SSP)

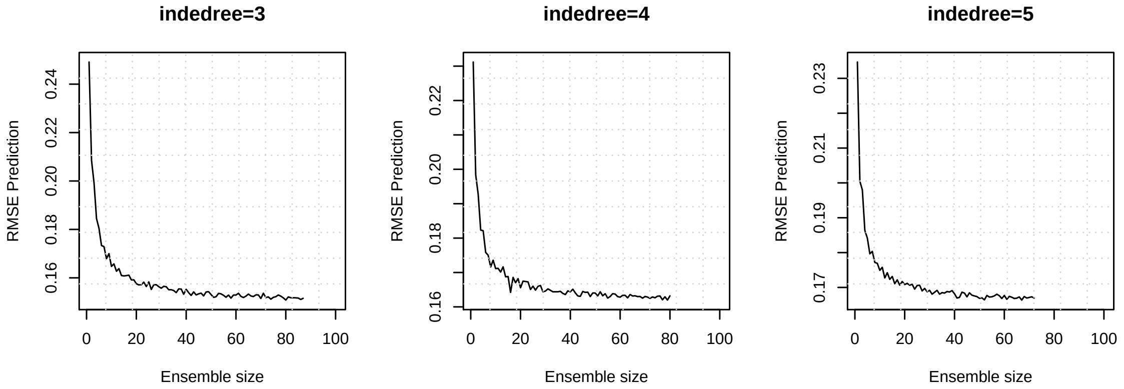

Figure 3: *Ensemble predictive skill depending on ensemble size for case study 2 (SSP).* The curve was computed by bootstrapping multiple $n_{\mathcal{M}}$ models from the available models, *i.e.* we sampled multiple realizations of the individual predictions for the same ensemble size and computed the average value. These curves converge asymptotically and show that the chosen ensemble size parameter is adequate. Equivalent predictions could have been obtained with smaller ensemble sizes.

1.4 Case study 3 (DREAMiS)

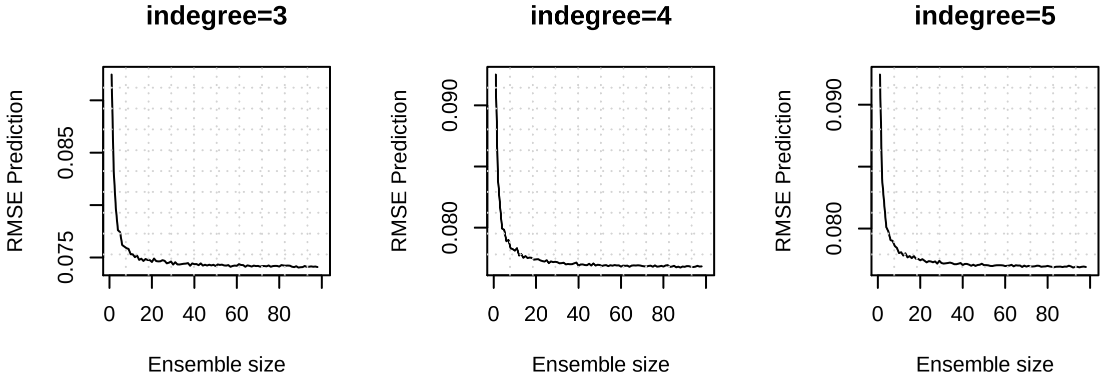

Figure 4: *Ensemble predictive skill depending on ensemble size for case study 3 (DREAMiS).* The curve was computed by bootstrapping multiple $n_{\mathcal{M}}$ models from the available models, *i.e.* we sampled multiple realizations of the individual predictions for the same ensemble size and computed the average value. These curves converge asymptotically and show that the chosen ensemble size parameter is adequate. Equivalent predictions could have been obtained with smaller ensemble sizes.

1.5 Case study 4a (DREAMBT20)

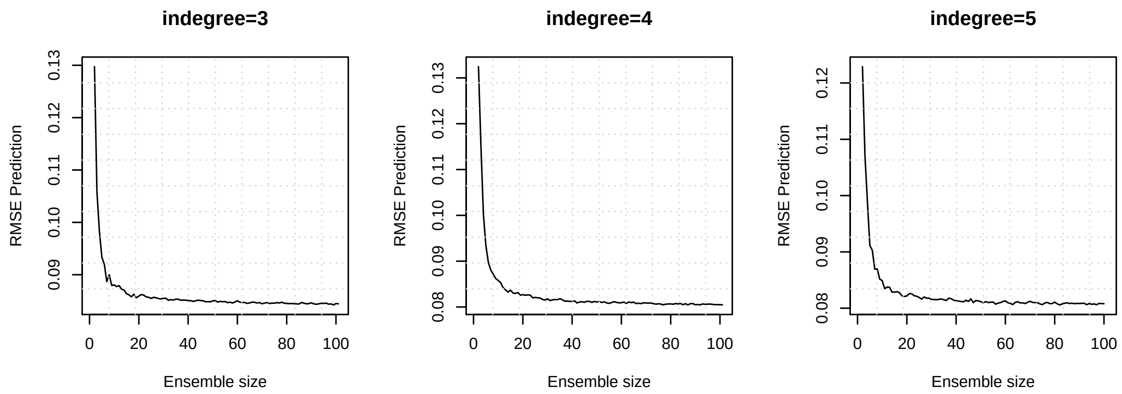

Figure 5: *Ensemble predictive skill depending on ensemble size for case study 4a (DREAMBT20).* The curve was computed by bootstrapping multiple $n_{\mathcal{M}}$ models from the available models, *i.e.* we sampled multiple realizations of the individual predictions for the same ensemble size and computed the average value. These curves converge asymptotically and show that the chosen ensemble size parameter is adequate. Equivalent predictions could have been obtained with smaller ensemble sizes.

1.6 Case study 4b (DREAMBT549)

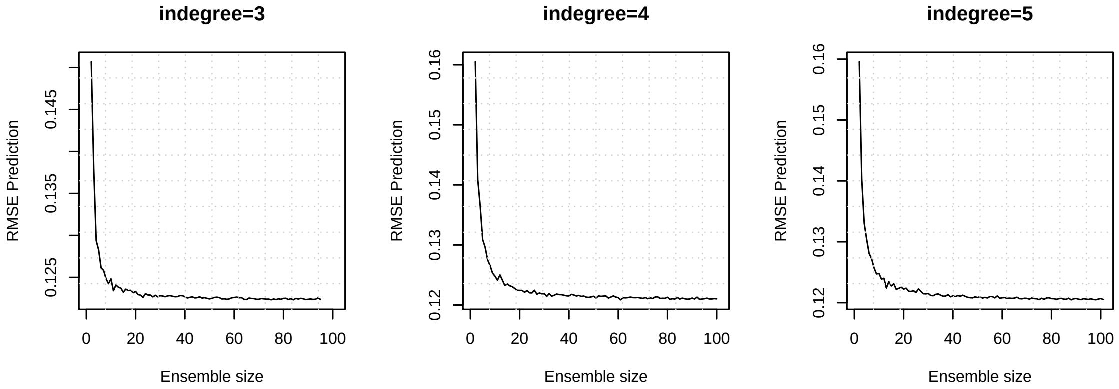

Figure 6: *Ensemble predictive skill depending on ensemble size for case study 4b (DREAMBT549).* The curve was computed by bootstrapping multiple $n_{\mathcal{M}}$ models from the available models, *i.e.* we sampled multiple realizations of the individual predictions for the same ensemble size and computed the average value. These curves converge asymptotically and show that the chosen ensemble size parameter is adequate. Equivalent predictions could have been obtained with smaller ensemble sizes.

1.7 Case study 4c (DREAMMCF7)

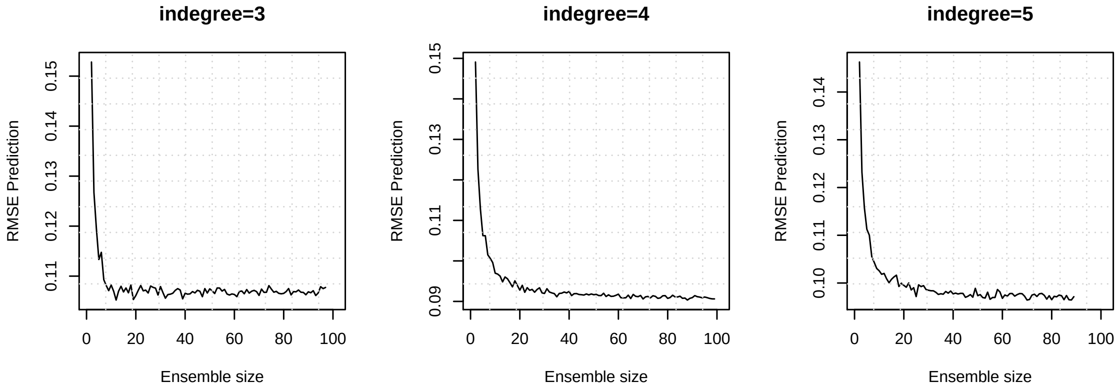

Figure 7: *Ensemble predictive skill depending on ensemble size for case study 4c (DREAMMCF7).* The curve was computed by bootstrapping multiple $n_{\mathcal{M}}$ models from the available models, *i.e.* we sampled multiple realizations of the individual predictions for the same ensemble size and computed the average value. These curves converge asymptotically and show that the chosen ensemble size parameter is adequate. Equivalent predictions could have been obtained with smaller ensemble sizes.

1.8 Case study 4d (DREAMUACC812)

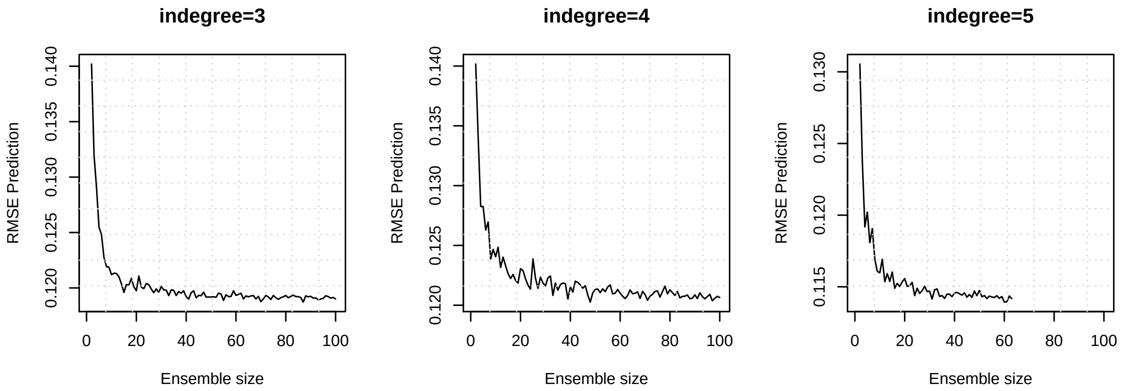

Figure 8: *Ensemble predictive skill depending on ensemble size for case study 4d (DREAMUACC812).* The curve was computed by bootstrapping multiple $n_{\mathcal{M}}$ models from the available models, *i.e.* we sampled multiple realizations of the individual predictions for the same ensemble size and computed the average value. These curves converge asymptotically and show that the chosen ensemble size parameter is adequate. Equivalent predictions could have been obtained with smaller ensemble sizes.
