# Supplementary material for: Data-driven reverse engineering of signaling pathways using ensembles of dynamic models
Source: PLoS Comput Biol. 2017 Feb 6;13(2):e1005379. doi: 10.1371/journal.pcbi.1005379 (PMC5319798; doi:10.1371/journal.pcbi.1005379)
Supplement: S5 Text — This curve was computed by bootstrapping multiple nℳ models from the available 100 models, i.e. we sampled multiple realizations of the ensemble network for the same ensemble size and computed the average value. (PDF) [file pcbi.1005379.s005.pdf]

S5 Text: AUPR score depending on ensemble size for different case-studies with and without applying model reduction

Data-driven reverse engineering of signaling pathways using ensembles of dynamic models

David Henriques, Alejandro F. Villaverde, Miguel Rocha, Julio Saez-Rodriguez, Julio R. Banga

January 17, 2017

Contents

1 Case study 1: AUPR score depending on ensemble size for different case-studies with and without applying model reduction 3
1.1 Case study 1a (MAPKp) 3
1.2 Case study 1b (MAPKf) 5
1.3 Case study 2 (SSP) 7
1.4 Case study 3 (DREAMiS) 9

List of Figures

1 AUPR score depending on ensemble size without model reduction for case study 1a (MAPKp) 3
2 AUPR score depending on ensemble size with model reduction for case study 1a (MAPKp) 4
3 AUPR score depending on ensemble size without model reduction for case study 1b (MAPKf) 5
4 AUPR score depending on ensemble size with model reduction for case study 1b (MAPKf) 6
5 AUPR score depending on ensemble size without model reduction for case study 2 (SSP) 7
6 AUPR score depending on ensemble size with model reduction for case study 2 (SSP) 8
7 AUPR score depending on ensemble size without model reduction for case study 3 (DREAMiS) 9
8 AUPR score depending on ensemble size with model reduction for case study 3 (DREAMiS) 10

1 Case study 1: AUPR score depending on ensemble size for different case-studies with and without applying model reduction

1.1 Case study 1a (MAPKp)

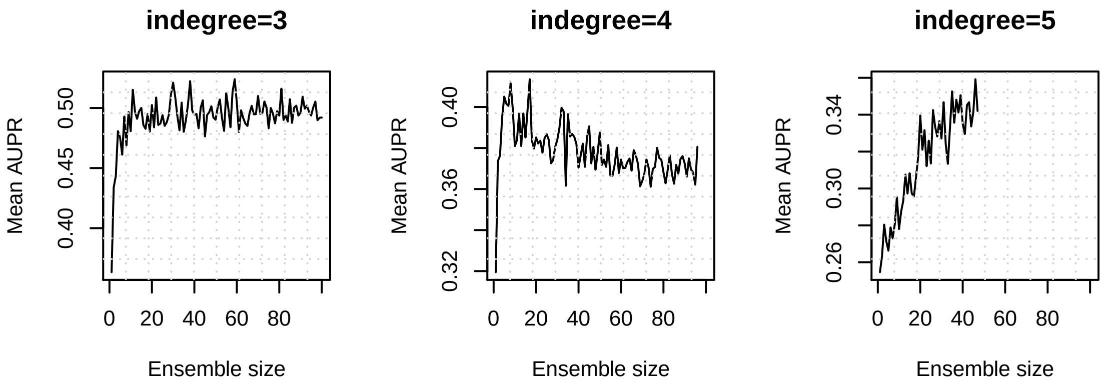

Figure 1:
AUPR score depending on ensemble size without model reduction for case study 1a (MAPKp). Descrition.This curve was computed by bootstrapping multiple $n_{\mathcal{M}}$ models from the available models, *i.e.* we sampled multiple realizations of the ensemble network for the same ensemble size and computed the average value.

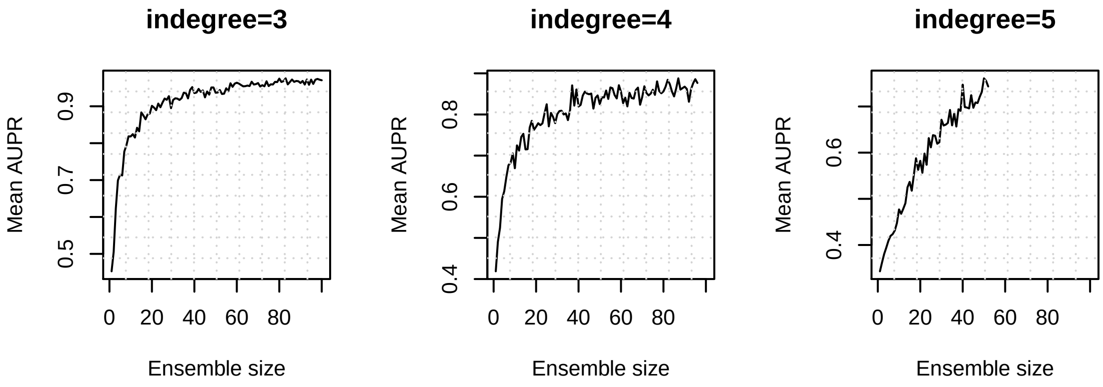

Figure 2:
AUPR score depending on ensemble size with model reduction for case study 1a (MAPKp). Descrition.This curve was computed by bootstrapping multiple $n_{\mathcal{M}}$ models from the available models, *i.e.* we sampled multiple realizations of the ensemble network for the same ensemble size and computed the average value.

1.2 Case study 1b (MAPKf)

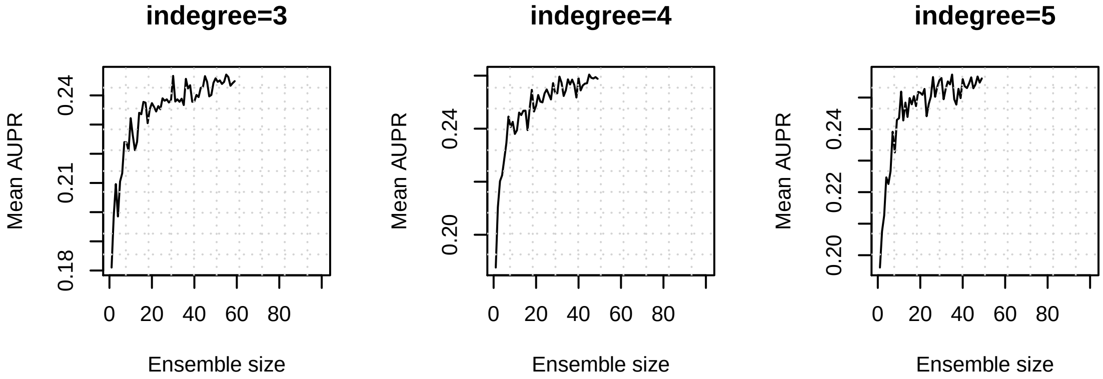

Figure 3:
AUPR score depending on ensemble size without model reduction for case study 1b (MAPKf). Descrition.This curve was computed by bootstrapping multiple $n_{\mathcal{M}}$ models from the available models, *i.e.* we sampled multiple realizations of the ensemble network for the same ensemble size and computed the average value.

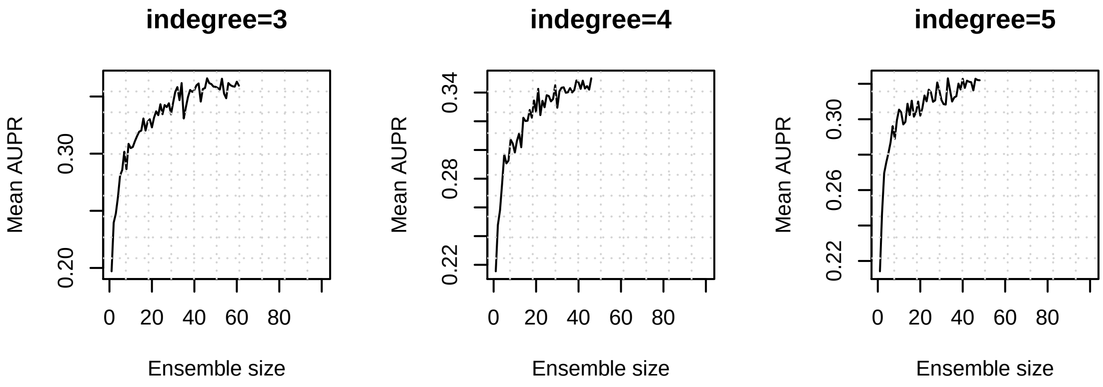

Figure 4:
AUPR score depending on ensemble size with model reduction for case study 1b (MAPKf). Descrition.This curve was computed by bootstrapping multiple $n_{\mathcal{M}}$ models from the available models, *i.e.* we sampled multiple realizations of the ensemble network for the same ensemble size and computed the average value.

1.3 Case study 2 (SSP)

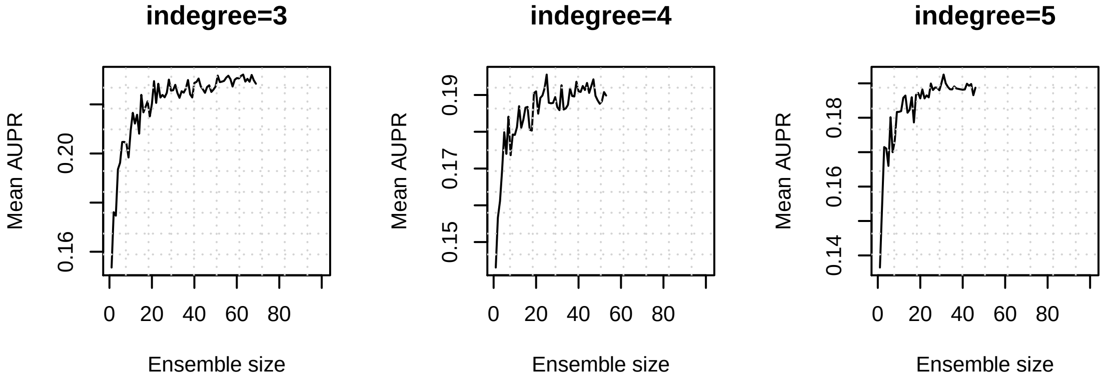

Figure 5:
AUPR score depending on ensemble size without model reduction for case study 2 (SSP). Descrition.This curve was computed by bootstrapping multiple $n_{\mathcal{M}}$ models from the available models, *i.e.* we sampled multiple realizations of the ensemble network for the same ensemble size and computed the average value.

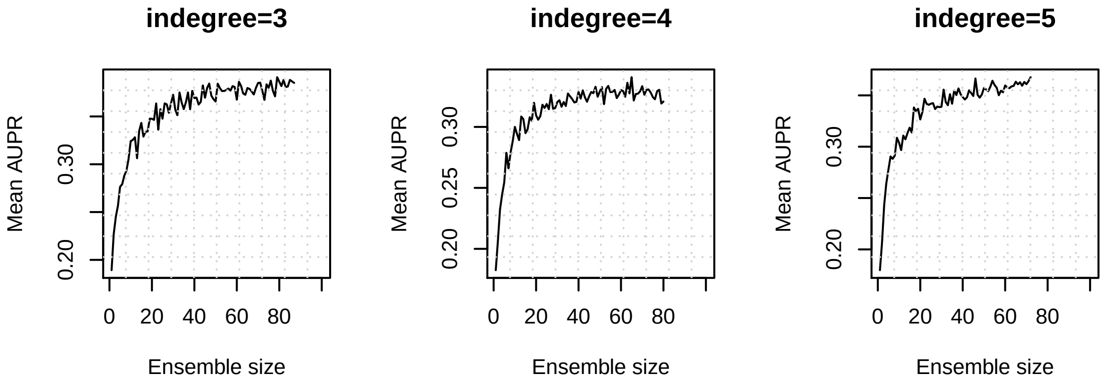

Figure 6:
AUPR score depending on ensemble size with model reduction for case study 2 (SSP). Descrition.This curve was computed by bootstrapping multiple $n_{\mathcal{M}}$ models from the available models, *i.e.* we sampled multiple realizations of the ensemble network for the same ensemble size and computed the average value.

1.4 Case study 3 (DREAMiS)

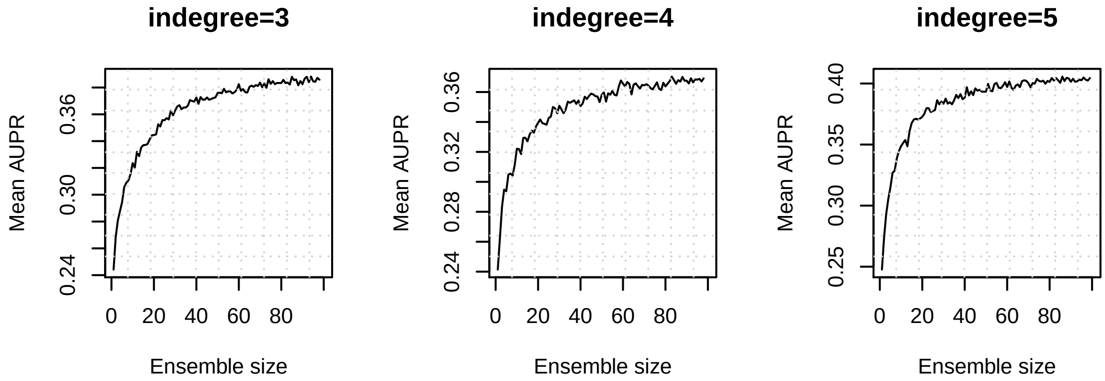

Figure 7:
AUPR score depending on ensemble size without model reduction for case study 3 (DREAMiS). Descrition.This curve was computed by bootstrapping multiple $n_{\mathcal{M}}$ models from the available models, *i.e.* we sampled multiple realizations of the ensemble network for the same ensemble size and computed the average value.

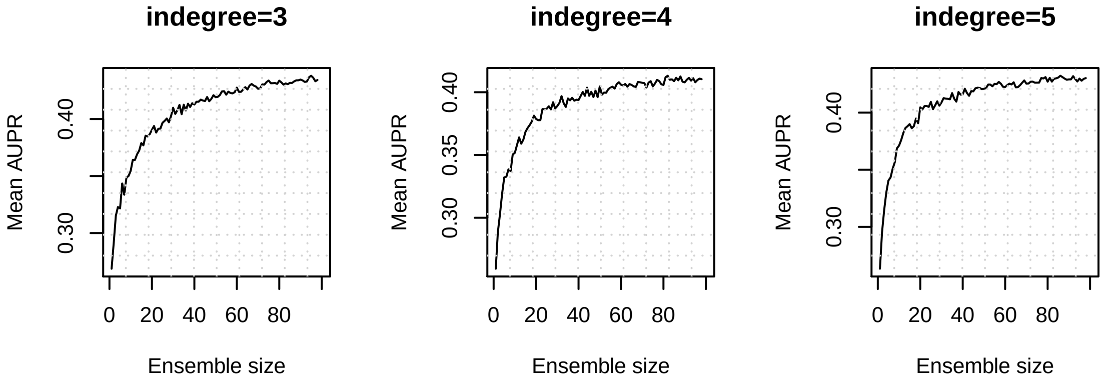

Figure 8:
AUPR score depending on ensemble size without model reduction for case study 3 (DREAMiS). Descrition.This curve was computed by bootstrapping multiple $n_{\mathcal{M}}$ models from the available models, *i.e.* we sampled multiple realizations of the ensemble network for the same ensemble size and computed the average value.
