# Supplementary material for: Data-driven reverse engineering of signaling pathways using ensembles of dynamic models
Source: PLoS Comput Biol. 2017 Feb 6;13(2):e1005379. doi: 10.1371/journal.pcbi.1005379 (PMC5319798; doi:10.1371/journal.pcbi.1005379)
Supplement: S6 Text — (PDF) [file pcbi.1005379.s006.pdf]

S6 Text: AUPR curves of different inference methods and case-studies

Data-driven reverse engineering of signaling pathways using ensembles of dynamic models

David Henriques, Alejandro F. Villaverde, Miguel Rocha, Julio Saez-Rodriguez, Julio R. Banga

January 17, 2017

Contents

1 AUPR curves for different algorithms and case-studies 3
1.1 Case study 1a (MAPKp) 3
1.2 Case study 1b (MAPKf) 4
1.3 Case study 2 (SSP) 5
1.4 Case study 3 (DREAMiS) 6

List of Figures

1 AUPR curves of different inference methods for case study 1a (MAPKp) 3
2 AUPR curves of different inference methods for case study 1b (MAPKf) 4
3 AUPR curves of different inference methods for case study 2 (SSP) 5
4 AUPR curves of different inference methods for case study 3 (DREAMiS) 6

1 AUPR curves for different algorithms and case-studies

1.1 Case study 1a (MAPKp)

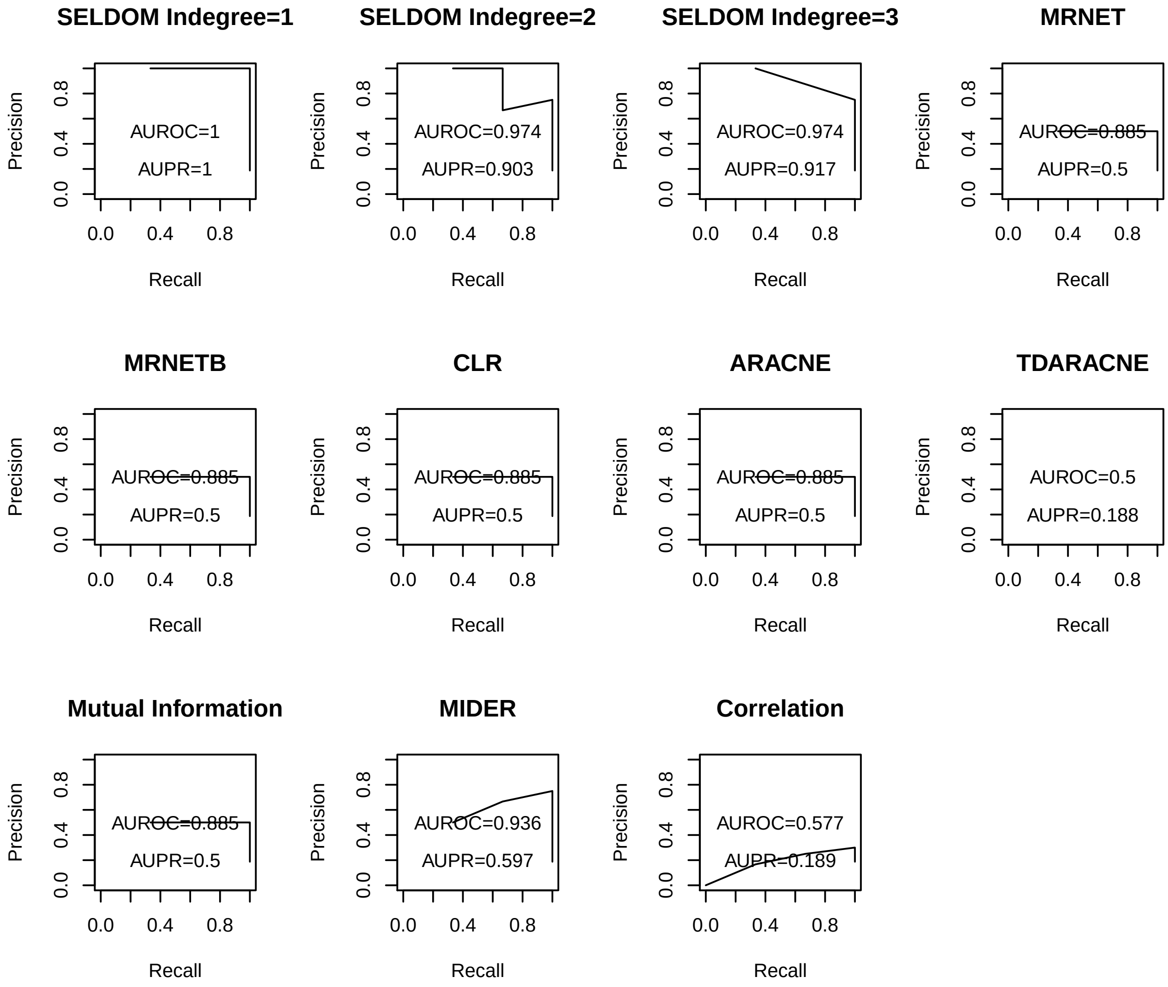

Figure 1: *AUPR curves of different inference methods for case study 1a (MAPKp).*

1.2 Case study 1b (MAPKf)

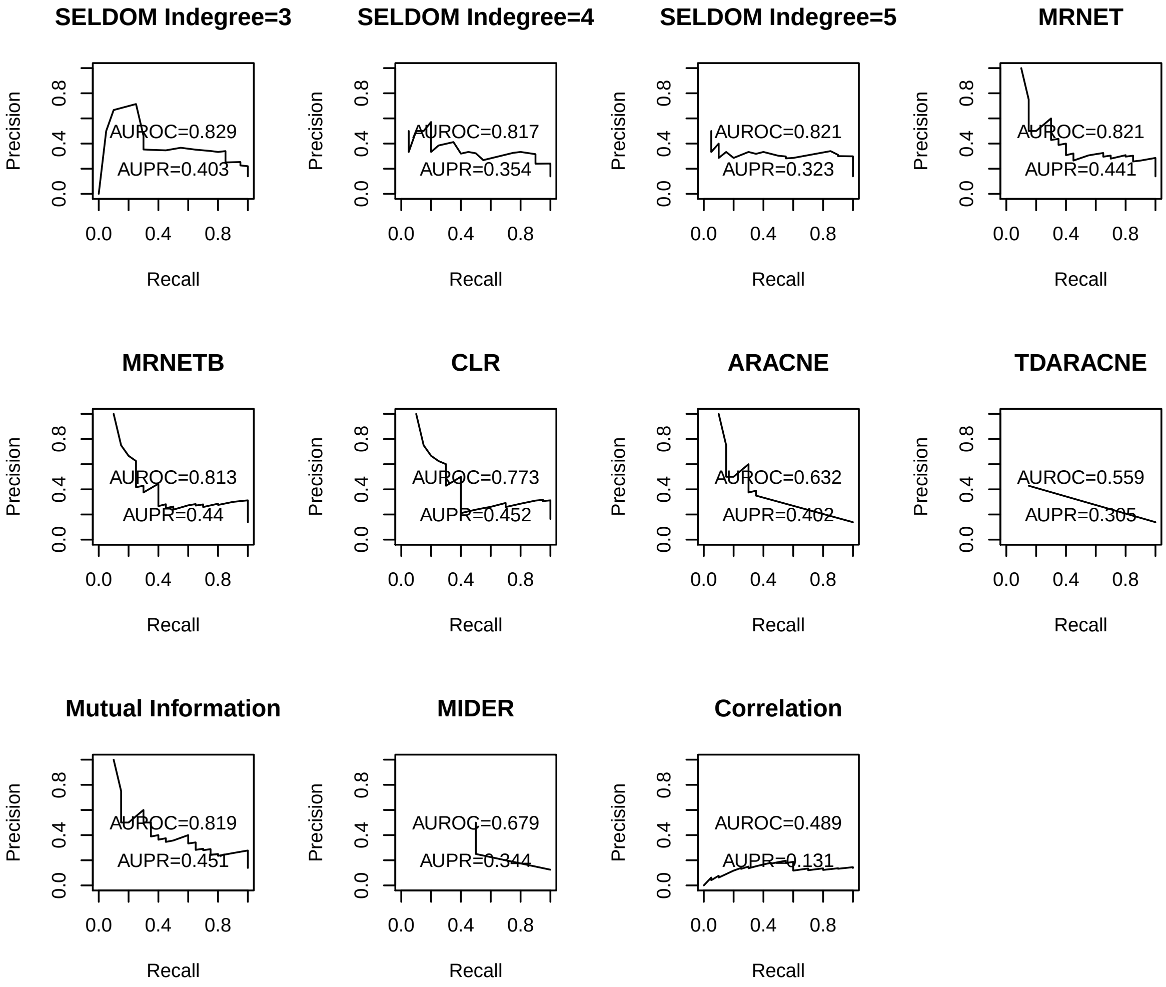

Figure 2: *AUPR curves of different inference methods for case study 1b (MAPKf).*

1.3 Case study 2 (SSP)

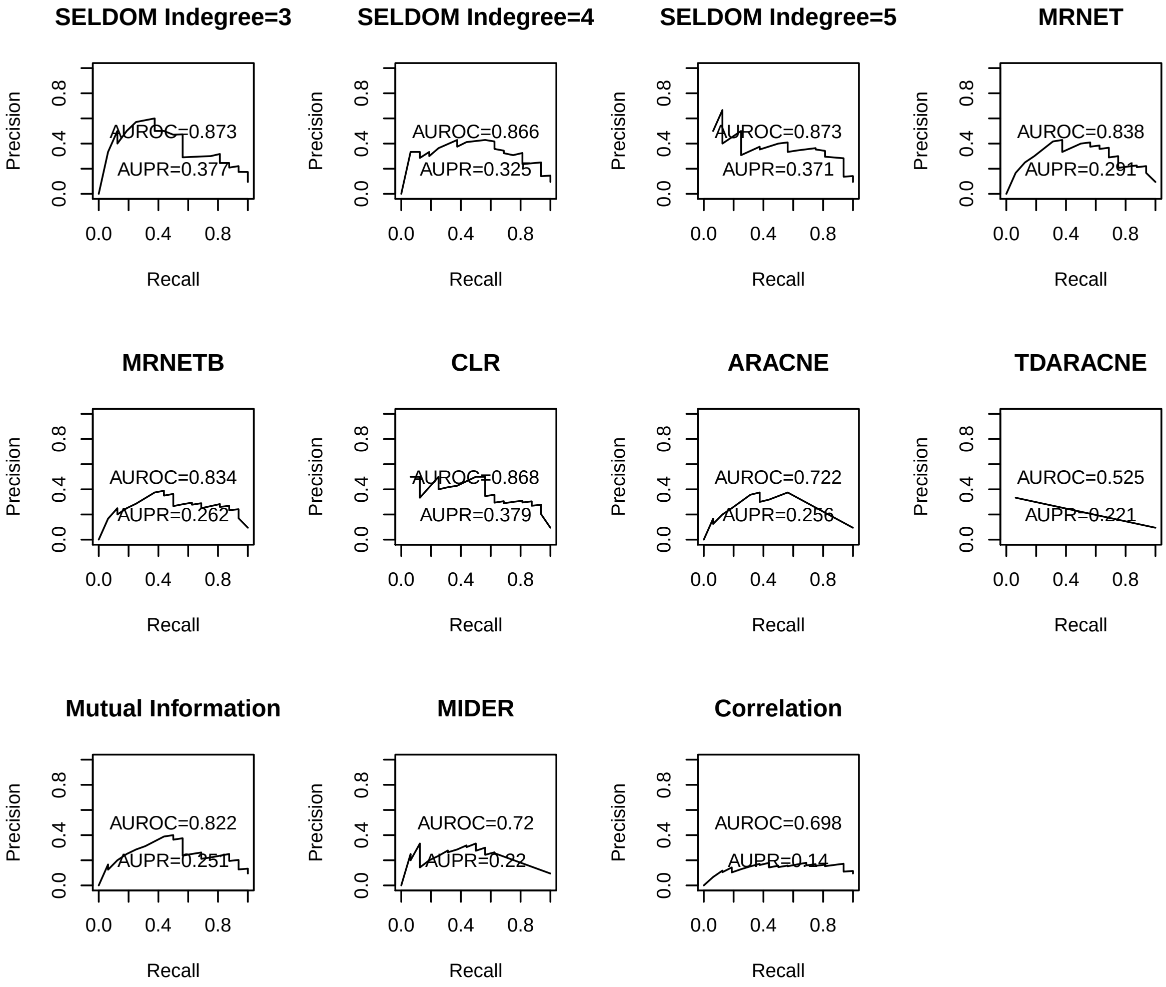

Figure 3: *AUPR curves of different inference methods for case study 2 (SSP).*

1.4 Case study 3 (DREAMiS)

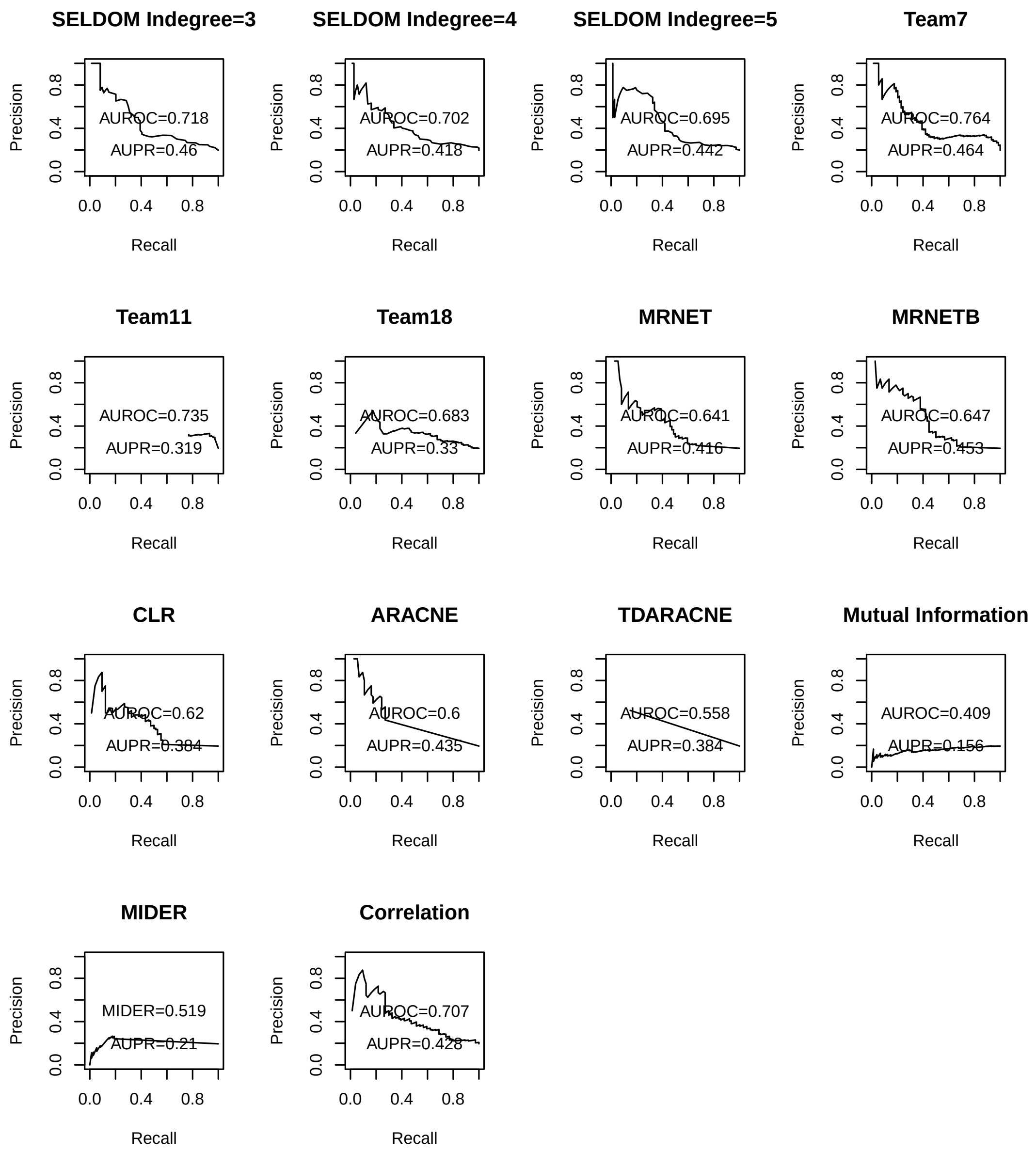

Figure 4: *AUPR curves of different inference methods for case study 3 (DREAMiS).*
