# Supplementary material for: Data-driven reverse engineering of signaling pathways using ensembles of dynamic models
Source: PLoS Comput Biol. 2017 Feb 6;13(2):e1005379. doi: 10.1371/journal.pcbi.1005379 (PMC5319798; doi:10.1371/journal.pcbi.1005379)
Supplement: S7 Text — (PDF) [file pcbi.1005379.s007.pdf]

S7 Text: Networks obtained with SELDOM for different cell-lines of HPN-DREAM experimental data sub-challenge

Data-driven reverse engineering of signaling pathways using ensembles of dynamic models

David Henriques, Alejandro F. Villaverde, Miguel Rocha, Julio Saez-Rodriguez, Julio R. Banga

January 17, 2017

Contents

1 Networks obtained with SELDOM for different cell-lines of the HPN-DREAM experimental data sub-challenge 3

List of Figures

1 Network for cell-line DREAMBT20 5
2 Network for cell-line DREAMBT549 5
3 Network for cell-line DREAMMCF7. 6
4 Network for cell-line DREAMUACC812. 6
5 Heatmap of the top 20 interactions found across all cell-lines. . . 7

List of Tables

1 Literature support of the 20 top interactions found across all cell-lines, part 1 8
2 Literature support for the 20 top interactions found across all cell-lines, part 2 9

1 Networks obtained with SELDOM for different cell-lines of the HPN-DREAM experimental data sub-challenge

In Figures 1 to 4 we show networks obtained with SELDOM for different cell-lines of the HPN-DREAM experimental data sub-challenge. SELDOM provides an ensemble network which is the average of all networks found by individual models after model reduction (see paper for more details). This is encoded in a adjacency matrix where each edge has a frequency score. To extract an interpretable network we built a network with edges with scores higher than 0,175 threshold. Sparser or denser networks could have been obtained by changing this threshold. The density of the edge color gives a sense of the frequency it appears in the models. Stimuli are colored in green and inhibitors in red. The stimuli FGF1 is sometimes inhibited and is therefore surrounded by a red line. All nodes in white color correspond to observed signals.

Figure 5 shows the relative score for the top 20 interactions found across all cell-lines. This score is the frequency that an interaction appears in the ensemble of inferred networks. Each trained model is based in a network that has undergone model reduction. Since these are the interactions for which SELDOM gives the highest support, we searched in pathway curated databases if they have been reported before to validate SELDOMs findings, we used OmniPath [5], a portal integrating dozens of high-quality manually curated data-bases. We retrieved from Omnipath the publications associated with those 20 interactions. Their PUBMED IDs are shown in Tables 1 and 2. From the set of 20 interactions, only 4 did not have associated publications according to OmniPath, suggesting that the interactions inferred from SELDOM are generally real. In contrast, this list of interactions provides a set of candidates for potentially unknown interactions:

1. S6 pS240 S244→S6 pS235 S236
2. MEK1 pS217 S221→BAD pS112
3. P70S6K pT389→BAD pS112
4. GSK3-alpha-beta pS21 S9→PRAS40 pT246

We further explored manually the literature for this list of interactions. In the case of interaction 1 (S6 pS240 S244→S6 pS235 S236), the S6 protein has multiple phospho-sites that are phosphorylated in a sequential manner [1], thus, according to the literature, this interaction can be regarded as a probable true positive. In the second interaction (MEK1 pS217 S221→BAD pS112), MEK has been reported as an indirect regulator of BAD. It has been shown that the phosphorylation of site S112 in BAD requires activation of the MAPK pathway and that inhibiting MEK with inhibitor PD98059 when RAS-RAF is active, blocks the phosphorylation of site S112 [2]. Thus, MEK is, at least indirectly, a regulator of BAD pS112, probably via ERK. In the case of interaction 3 (p70S6K

pT389→BAD pS112), it has been identified that p70S6K is a BAD specific kinase in site S136 (but not S112, which is a substrate of p70SK6) [3]. Hence, this is probably a false positive. Finally, we could not find support for the interaction GSK3alphabeta pS21 S9→PRAS40 pT246 although the phosphorylation state of GSK3beta and PRAS40 depends on AKT [4]. Hence, it could could be that this interaction is a false positive or a new, yet uncharacterized link. Thus, this link is an example of a candidate obtained from applying SELDOM that would be worth to further study experimentally. Collectively, these results show how SELDOM, while in some cases find false positives, in other recovers known biology, and it is also able to point at candidates for further study, hence being a useful tool to guide experiments to gain biological insight.

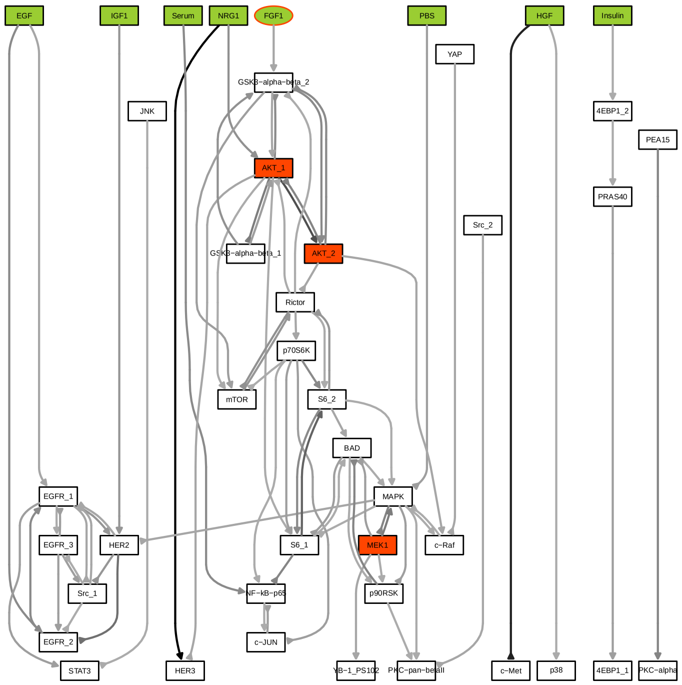

Figure 1: Network for cell-line DREAMBT20.

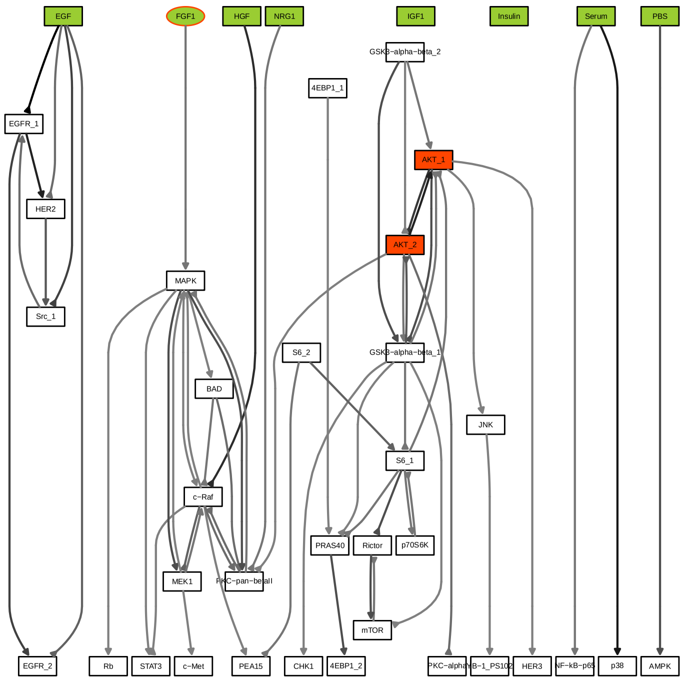

Figure 2: Network for cell-line DREAMBT549.

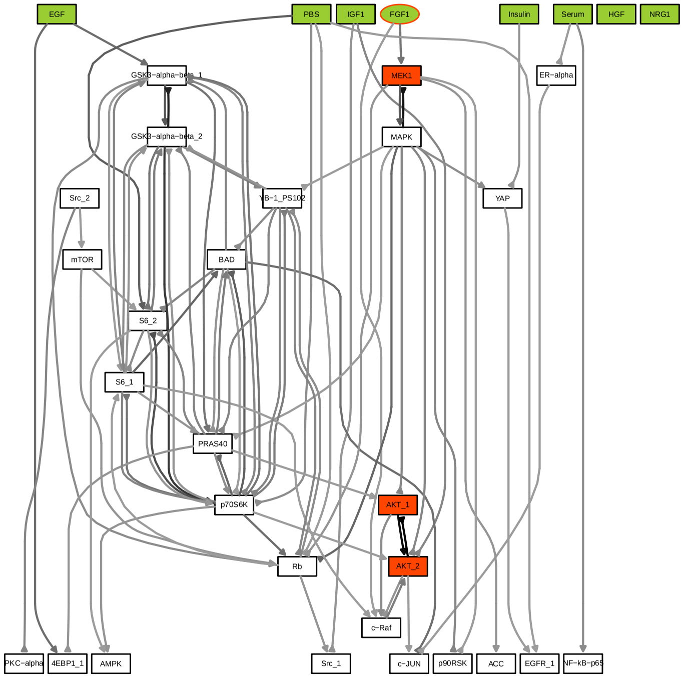

Figure 3: Network for cell-line DREAMMCF7.

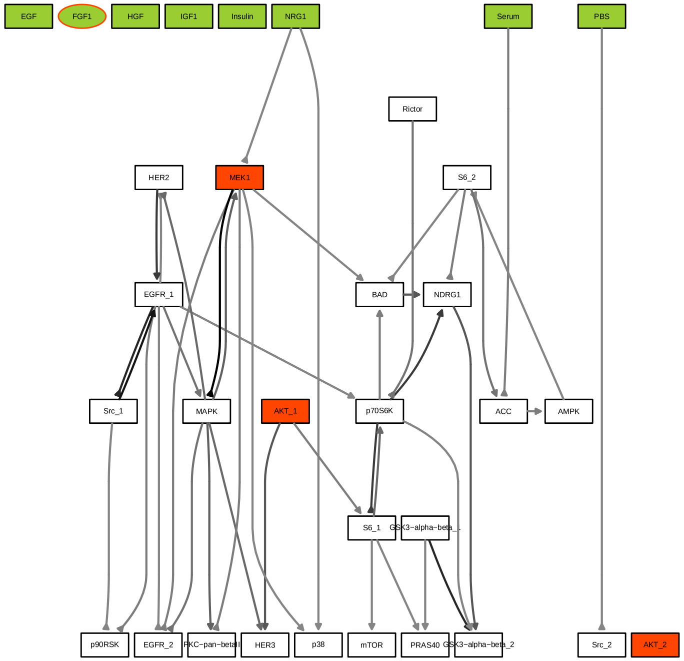

Figure 4: Network for cell-line DREAMUACC812.

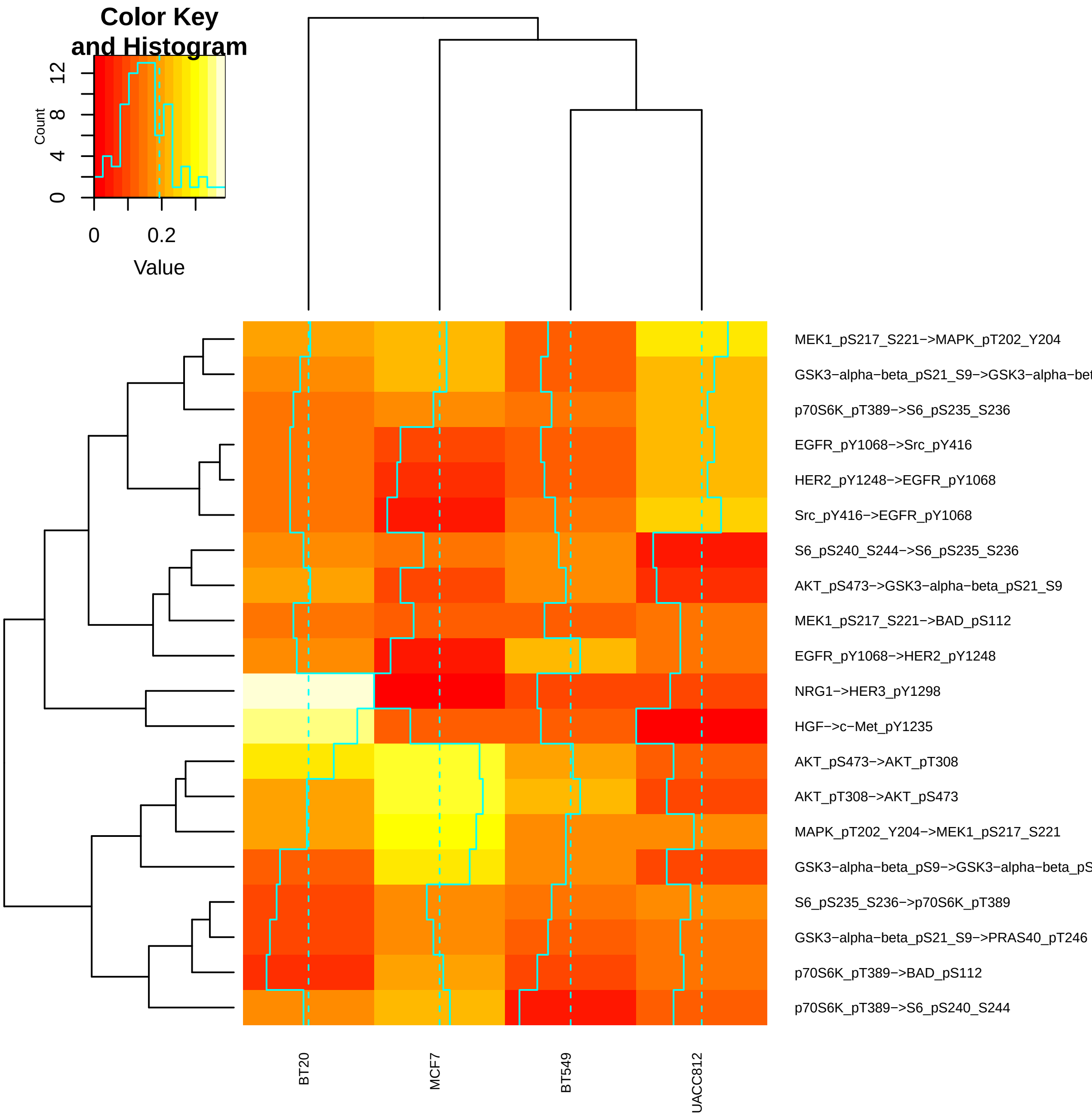

Figure 5: Heatmap of the top 20 interactions found across all cell-lines.

Source ID	Source Name	Target ID	Target Name	Direction	PubMed ID
Q02750	MEK1	P28482	MAPK	directed	18083107; 19053285; 19494114; 20068231; 21842857; 16887817; 19534553; 17906627; 15935618; 16497976; 11971971; 11251083; 12270934; 18578522; 15592455; 16484222; 17016520; 19901323; 18452278; 12644307; 15951569; 12388769; 19690332; 18691976; 19651622; 18707149; 11522288; 16153436; 19369195; 19415658; 18669648; 8226933; 17389395; 17081983; 17192257; 11522789; 16094384; 9166761
P49840	GSK3A	P49840	GSK3A	directed	15020233; 18088087; 18701488; 19415658; 17389395; 19664994; 17192257; 19413330; 15592455; 14570592; 18452278; 18767875
P49841	GSK3B	P49841	GSK3B	directed	18701488; 14570592; 20331603; 11440715
P23443	RPS6KB1	P62753	RPS6	directed	1939282; 17360704; 8440735; 15471950; 20227368; 15809305; 7705100; 18423201; 20464435; 15358595; 24396066; 23899556 S235; S236
P00533	EGFR	P12931	Src	directed	21278788; 25402006; 24189400; 20333651; 23175185; 21268077; 10358079; 17158602; 10075741; 14963038; 10777553; 10971656; 20935677; 24658140; 11983694; 8845374; 21573184; 17284441
P04626	HER2	P00533	EGFR	directed	18691976; 9363897; 16829981; 8816440; 18669648; 16799092; 17389395; 10635327; 17016520
P12931	Src	P00533	EGFR	directed	21787862; 25958313; 3138233; 1956339; 16729043; 7805884; 14530278; 2115882; 10075741; 20407013; 16943190; 15660394; 1651323; 16799092; 16505275; 15618223; 17016520; 18234969; 9777370; 14764607; 15951569; 12397069; 25347738; 23246962; 11983694; 18789131; 15282306; 15592458; 14963038; 19377510; 2543683; 26494466; 18669648; 12063263; 7505275; 17389395; 16849545; 8845374; 16936701; 1309762; 16461772; 16862179
P62753	RPS6	P62753	RPS6		
P31749	AKT	P49840	GSK3A	directed	8524413; 11749387; 12054501; 18691976; 11035810; 17192257; 11884598; 19413330; 19369195

Table 1: Literature support of the 20 top interactions found across all cell-lines, part 1

Source ID	Source Name	Target ID	Target Name	Direction	PubMed ID
P31749	AKT	P49841	GSK3B	directed	14744935; 10716737; 20028854; 18199533; 16806104; 19254569; 23352416; 21996745; 25714023; 19369195; 19179806; 17041888; 17908691; 22278060; 16188907; 16115820; 15126379; 16153639; 12228224; 20506302; 24705253; 20352103; 21237163; 14561750; 14990992; 18209096; 12890758; 20186153; 18201972; 11340086; 7980435; 14713298; 15132987; 18669648; 25896507; 15735754; 15457186; 16971387; 19416964; 21775285; 20371716; 15950778; 15255949; 19116273; 12052830; 11583116; 18451303; 15561713; 21445305; 12900420; 11956225; 23851495; 26219398; 15867245; 14988390; 15467740; 16546978; 23428800; 24981175; 19472218; 21217772; 20637111; 19797085; 20548028; 12200436; 21868755; 19920184; 11278246; 20224723; 11563975; 19060929; 14550568; 12167717; 12054501; 9373175; 15818404; 8524413; 18577697; 8250835; 17724478; 16601140; 12649200; 12764143; 17693255; 17495324; 15805282; 23552696; 11035810; 20101232; 14633703; 16987514; 18167338; 15808505; 12167628; 15799972; 21914810; 15878878; 15208673; 24879152; 18216173; 17008323; 21954288; 21229324; 16959574; 25619831; 11520785; 16275989; 12522140; 11495916; 11254732; 16123357; 20233866; 16407243; 11331582; 25714029; 18593907; 18691976; 11289110; 11809746; 17623045; 19064993; 16039586; 11389054; 21177249; 17192257; 21308747; 25584893; 20190034; 11579131; 18757413; 19004823; 16987962; 18387957; 22080480; 15684397; 11779850; 16484495; 21441927; 8985174; 21532623; 21145761; 16476742; 12871932; 19996208; 19878439; 14660586; 21296133
Q02750	MEK1	Q92934	BAD		
P00533	EGFR	P04626	HER2	directed	11500516; 15695410; 17389395; 17426253; 1706616; 11279155; 12354693
Q02297	NRG1	P21860	HER3	directed	7477375; 7514177
P14210	HGF	P08581	MET	directed	8380735; 1846706
P31749	AKT	P31749	AKT	directed	22706160; 16549426; 15998322
P31749	AKT	P31749	AKT	directed	22706160; 16549426; 15998322
P28482	MAPK	Q02750	MEK1	directed	10567369; 7873542; 12594221; 9351825; 8039496; 15466476; 14993270; 8157000; 7624324
P49840	GSK3A	P49840	GSK3A	directed	15020233; 18088087; 18701488; 19415658; 17389395; 19664994; 17192257; 19413330; 15592455; 14570592; 18452278; 18767875
P49841	GSK3B	P49841	GSK3B	directed	18701488; 14570592; 20331603; 11440715
P62753	RPS6	P23443	RPS6KB1	undirected	21460630; 11438723; 17693255; 16810323
P49840	GSK3A	Q96B36	PRAS40		
P49841	GSK3B	Q96B36	PRAS40		
P23443	RPS6KB1	Q92934	BAD		
P23443	RPS6KB1	P62753	RPS6	directed	1939282; 17360704; 8440735; 15471950; 20227368; 15809305; 7705100; 18423201; 20464435; 15358595; 24396066; 23899556

Table 2: Literature support for the 20 top interactions found across all cell-lines, part 2

References

[1] Anne Biever, Emmanuel Valjent, and Emma Puighermanal. Ribosomal protein s6 phosphorylation in the nervous system: From regulation to function. *Frontiers in molecular neuroscience*, 8, 2015.

[2] Xianjun Fang, Shuangxing Yu, Astrid Eder, Muling Mao, Robert C Bast, Douglas Boyd, and Gordon B Mills. Regulation of bad phosphorylation at serine 112 by the ras-mitogen-activated protein kinase pathway. *Oncogene*, 18(48), 1999.

[3] Hisashi Harada, Jens S Andersen, Matthias Mann, Naohiro Terada, and Stanley J Korsmeyer. p70s6 kinase signals cell survival as well as growth, inactivating the pro-apoptotic molecule bad. *Proceedings of the National Academy of Sciences*, 98(17):9666–9670, 2001.

[4] Satoshi Rokutanda, Takashi Fujita, Naoko Kanatani, Carolina A Yoshida, Hisato Komori, Wenguang Liu, Akio Mizuno, and Toshihisa Komori. Akt regulates skeletal development through gsk3, mtor, and foxos. *Developmental biology*, 328(1):78–93, 2009.

[5] Dénes Türei, Tamás Korcsmáros, and Julio Saez-Rodriguez. Omnipath: guidelines and gateway for literature-curated signaling pathway resources. *Nature methods*, 13(12):966–967, 2016.
